# CDK4 Mediates Cisplatin Resistance in Renal Cell Carcinoma (RCC) Cells by Regulating the ASH1L-CTR1 Axis

**DOI:** 10.32604/or.2026.073934

**Published:** 2026-05-21

**Authors:** Wenjian Zeng, Xianglong Li, Hao Cai, Qingyu Zhou, Shuangshuang Sun, Pingping Li, Sunlong Li, Zhi Chen

**Affiliations:** 1 Department of Oncology, the Second Affiliated Hospital of Wenzhou Medical University, Wenzhou, China; 2 Department of Chemotherapy, the Second Affiliated Hospital of Wenzhou Medical University, Wenzhou, China

**Keywords:** Renal cell carcinoma, cisplatin resistance, cyclin-dependent kinase 4 (CDK4), ASH1-Like Histone Lysine Methyltransferase (ASH1L), copper transporter 1 (CTR1), molecular mechanism

## Abstract

**Objectives:** Cisplatin resistance is a major obstacle in the treatment of renal cell carcinoma (RCC), severely compromising therapeutic efficacy and patient prognosis. This study aimed to clarify the role and molecular mechanism of cyclin-dependent kinase 4 (CDK4) in cisplatin resistance of RCC. **Methods:** Immunohistochemistry (IHC) was used to detect the expression of CDK4 in cisplatin-resistant RCC tissues. In RCC cells and their drug-resistant sublines, CDK4 overexpression/knockdown assays were performed to evaluate the effects on cisplatin resistance and malignant progression. An *in vivo* model was established, to verify the *in vivo* function of CDK4. Transcriptome sequencing (RNA-seq), Cleavage Under Targets and Tagmentation (CUT&Tag), chromatin immunoprecipitation (ChIP) and dual-luciferase reporter assays were applied to elucidate the downstream regulatory mechanism of CDK4. **Results:** CDK4 was highly expressed in Tumor-Drug Resistant (Tumor-DR) tissues, which was significantly correlated with poor patient prognosis. CDK4 overexpression enhanced cisplatin resistance and malignant phenotypes of RCC cells, whereas CDK4 knockdown reversed these effects and sensitized cells to cisplatin. *In vivo* experiments confirmed that CDK4 promoted cisplatin resistance and tumor growth of A498 cells, and this effect was validated under both CIS and GEM intervention. Mechanistically, CDK4 directly bound to the promoter region of the ASH1L (ASH1-Like Histone Lysine Methyltransferase) gene to promote its transcription; by upregulating ASH1L, CDK4 inhibited the expression of copper transporter 1 (CTR1), thereby mediating cisplatin resistance. Targeted inhibition of CDK4 or ASH1L enhanced the cisplatin sensitivity of RCC cells. **Conclusions:** This study identifies the critical role of the CDK4-ASH1L-CTR1 axis in cisplatin resistance of RCC.

## 1 Introduction

Renal cell carcinoma (RCC), as a common malignant tumor of the urinary system, shows an increasing incidence trend worldwide [1]. Although surgical resection is the main treatment for early-stage RCC, chemotherapy still plays an irreplaceable role in advanced or metastatic RCC [1,2,3,4]. Cisplatin, as a broad-spectrum chemotherapeutic drug, exerts its anti-tumor effect by binding to DNA to form cross-links and interfering with DNA replication and transcription processes [5,6,7]. However, in clinical treatment, the resistance of RCC to cisplatin is becoming increasingly serious, which greatly limits the clinical efficacy of cisplatin and has become a key problem to be solved urgently in the field of RCC treatment [8,9].

CDK4 (Cell division cycle 2-like kinase 4) is a member of the serine/threonine protein kinase family. Proteins of this family play crucial roles in various cellular physiological processes such as cell cycle regulation and RNA splicing [10,11,12,13]. In recent years, more and more studies have shown that CDK family proteins are closely related to tumor occurrence and development. For example, in tumors such as breast cancer and lung cancer, CDK proteins are involved in the regulation of tumor cell proliferation, apoptosis, and metastasis [14,15,16,17]. However, until now, the specific role and related molecular mechanism of CDK4 in RCC, especially in cisplatin resistance, have not been fully elucidated.

In the study of tumor resistance mechanisms, abnormal gene transcription regulation is one of the important reasons leading to tumor cell resistance. ASH1L (Absent, small, or homeotic-like 1), as a histone methyltransferase [18,19], can regulate the transcriptional expression of downstream genes by methylating histones, thereby participating in various biological processes of cells [20,21]. Existing studies have shown that ASH1L is abnormally expressed in some tumors and is related to the malignant phenotype and drug resistance of tumors [22,23]. CTR1 (Copper Transporter 1) is a copper transporter and also participates in the uptake of cisplatin in cells [24,25,26]. Studies have shown that the expression level of CTR1 is closely related to the sensitivity of tumor cells to cisplatin. The increased expression of CTR1 often enhances the uptake of cisplatin by cells, thereby increasing the sensitivity of cells to cisplatin, and *vice versa*, leading to drug resistance [27,28,29]. Beretta et al. further confirmed this by comparing multiple pairs of cisplatin-sensitive and resistant cell lines, showing that CTR1 expression is positively correlated with intracellular cisplatin accumulation and chemosensitivity [30]. Recent studies have shown that the function of CDK4 is not limited to cell cycle regulation: in various tumor models, CDK4 can translocate to the nucleus, bind to specific transcription factors, and directly target gene promoter regions to participate in transcriptional regulation, confirming its transcriptional regulatory potential beyond the scope of traditional cell cycle regulation [12,13]. As a histone methyltransferase, ASH1L can not only regulate the expression of tumor drug resistance-related genes through histone modification and mediate the resistance of tumor cells to chemotherapeutic drugs, but also has been observed to interact with copper metabolism-related proteins in studies, suggesting that it may be involved in the regulation of copper transporter family molecules [19]. Consistent with this, Du et al. verified that ASH1L can regulate protein-protein interaction networks to participate in membrane transport pathways in tumor cells [19], further supporting the potential regulatory relationship between ASH1L and copper transporters such as CTR1. Meanwhile, as a key copper transporter mediating the intracellular uptake of cisplatin, CTR1 expression level is closely related to the cisplatin sensitivity of cancer cells, and upregulating CTR1 expression can effectively reverse the drug-resistant phenotype of cells [27]. Notably, CDK4 has been reported to exert transcriptional regulatory functions beyond cell cycle control: Li et al. demonstrated that CDK4 interacts with SMYD2 to regulate gene transcription, which is involved in tubulin methylation and ciliogenesis [30], providing direct evidence for CDK4 to target gene promoters and mediate transcriptional regulation. Combining the aforementioned transcriptional regulatory ability of CDK4, the regulatory tendency of ASH1L on transporter molecules, and the core role of CTR1 in cisplatin resistance of renal cancer cells, we propose the hypothesis that CDK4 may mediate cisplatin resistance in A498-DR cells by regulating the transcription of ASH1L [31], thereby affecting the expression of CTR1.

Based on the above research background, this study hypothesizes that CDK4 may mediate cisplatin resistance in A498-DR cells by regulating the transcription of ASH1L and then affecting the expression of CTR1. To verify this hypothesis, we systematically investigated the role and molecular mechanism of CDK4 in cisplatin resistance of A498-DR cells through clinical sample analysis, cell function experiments, animal experiments, and a series of molecular biology techniques, aiming to provide new potential targets and theoretical basis for the treatment of RCC. At the same time, gemcitabine (GEM), as another anti-tumor drug, was also used in related intervention experiments to further explore the possibility of treatment strategies such as combination therapy.

## 2 Materials and Methods

### 2.1 Materials

Cell Source: The A498 (HTX2237) and RCC4 (HTX2897) were purchased from Otwo Biotech Inc. (Shenzhen, China). All cells were cultured in Dulbecco’s Modified Eagle’s Medium (DMEM) (Gibco, Cat. No.: 11965092, Waltham, MA, USA) supplemented with 10% fetal bovine serum (FBS, Gibco, Cat. No.: 10099-141, Waltham, MA, USA) and 1% penicillin-streptomycin (Gibco, Cat. No.: 15140-122, Waltham, MA, USA) at 37°C in a humidified incubator containing 5% CO_2_. Cells were routinely tested for mycoplasma contamination using a PCR-based detection kit (Thermo Fisher Scientific, Cat. No.: 4460623, Waltham, MA, USA) and confirmed to be mycoplasma-free. All cell lines were authenticated by short tandem repeat (STR) profiling at Otwo Biotech Inc; The nude mice were purchased from Charles River Laboratories (Beijing, China). Cycloheximide (CHX) was obtained from MedChemExpress (Cat. #HY-12320, Monmouth Junction, NJ, USA). Bafilomycin A1 (Baf-A1) was purchased from MedChemExpress (Cat. No. HY-100558, Monmouth Junction, NJ, USA). Crystal Purple was purchased from Solarbio (#G1063, Beijing, China). Cell Counting Kit-8 (CCK-8) was purchased from MedChemExpress (MCE, #HY-K0301, Monmouth Junction, NJ, USA) with the catalog number HY-K0301. A total of 197 renal cancer issue samples were included in this study, all of which were derived from the Second Affiliated Hospital of Wenzhou Medical University. Among them, there were 15 cases of normal renal tissues, 153 cases of cisplatin-sensitive RCC tissues (Tumor group), and 29 cases of cisplatin-resistant RCC tissues (Tumor-DR group). (The diagnostic criteria for cisplatin resistance mainly referred to the “Evaluation Criteria for the Efficacy of Solid Tumors (RECIST 1.1)” and clinical practice.) All cases were independently diagnosed and confirmed by three experienced pathologists. All patients included in this study provided written informed consent prior to the collection of clinical tissue samples. The consent form clearly stated the purpose of the study, the use of samples for scientific research, potential risks, and the right to withdraw from the study at any time without affecting medical treatment. All patients fully understood the study protocol and voluntarily signed the consent form, which was archived by the Second Affiliated Hospital of Wenzhou Medical University for long-term preservation. This study was conducted in strict accordance with the principles of the Declaration of Helsinki (2013 revision) and relevant ethical guidelines for medical research involving human subjects. All procedures related to human tissue samples and clinical data were reviewed and approved to ensure the protection of patients’ privacy, autonomy, and rights.

Ethics of Animal Experiments, Ethics Approval Number: 2025-KA-112-031, Issuing Ethics Committee: Ethics Committee of the Second Affiliated Hospital of Wenzhou Medical University. Ethics of human tissue samples, Ethics Approval Number: wyZw 2023-0700, Issuing Ethics Committee: Ethics Committee of the Second Affiliated Hospital of Wenzhou Medical University. All experimental protocols involving human tissues and animals were reviewed and approved by the above ethics committee. The study strictly followed the approved procedures to ensure compliance with ethical and legal requirements. Written informed consent was obtained from all patients or their legal guardians prior to the collection of human tissue samples, in strict compliance with the ethical guidelines of the committee and the Declaration of Helsinki.

### 2.2 Construction of Drug-Resistant Cell Lines

In the initial induction phase, parental A498 and RCC4 cells, which were cultured to 60% confluence in the logarithmic growth phase, were seeded at a density of 1 × 10^6^ cells per 10 cm culture dish and respectively inoculated into complete medium. (DMEM (Catalog No.: 11965092, Gibco)/RPMI1640 (Catalog No.: 27016021, Gibco) + 10% Fetal Bovine Serum (FBS, Catalog No.:10099158, Gibco) + 1% antibiotics (Catalog No.: 15140148, Gibco, Waltham, MA, USA) containing low-concentration cisplatin (0.5 μg/mL for A498; 0.8 μg/mL for RCC4, MedChemExpress, Catalog No.: HY-17394, Monmouth Junction, NJ, USA), and cultured in a constant temperature environment at 37°C with 5% CO_2_. The initial concentration was determined based on pre-experiment results to ensure the cell survival rate was maintained at 40%–50% and to avoid direct cell death caused by the drug. During the concentration increment and passage process, the medium containing cisplatin at the same concentration was replaced every 3 days. After the cells adapted (survival rate recovered to over 80%, approximately 7–10 days), the drug concentration was gradually increased according to the cisplatin concentration gradient (A498: 0.5→1.0→2.0→4.0→6.0 μg/mL; RCC4: 0.8→1.5→3.0→5.0→7.0 μg/mL), with an adaptation period of 10–14 days for each concentration gradient. The limiting dilution method (1–2 cell per well) was used throughout the process to maintain cell monoclonality and prevent contamination by heterogeneous cells. When the cells could grow stably in medium containing 6.0 μg/mL (for A498) and 7.0 μg/mL (for RCC4) cisplatin (MedChemExpress, Catalog No.: HY-17394), the successful construction of the drug-resistant cell lines was confirmed, and they were named A498-DR and RCC4-DR, respectively. For subsequent experiments, drug-resistant cells passaged within 20 generations were used to ensure the stability of the drug-resistant phenotype.

### 2.3 Immunohistochemistry

Tissue (The section thickness was set at 4 μm, the sample source was defined as surgically resected RCC tissues [Ethics Approval Number: wyZw 2023-0700], and the tissue fixation was performed with 4% paraformaldehyde for 24 h at 4°C.) sections were prepared. These sections were subjected to conventional dewaxing treatment and rinsed with water, three times each time with Phosphate-Buffered Saline (PBS) buffer (Gibco, #C10010500BT, Waltham, MA, USA) for 5 min. To reduce non-specific background staining caused by endogenous peroxidase, the sections were incubated in 3% hydrogen peroxide solution for 10 min, followed by another 3 washes with PBS buffer (Gibco, #C10010500BT) for 5 min each time. Then, 5% normal goat serum (Gibco, Catalog No.: 16210064) was added dropwise and incubated at room temperature for 15 min to block non-specific binding sites. The serum was poured off without washing, Antigen retrieval was performed using the microwave heating method. Sections were immersed in 0.01 mol/L citrate buffer (pH 6.0) and heated in a microwave oven for 15 min, followed by natural cooling to room temperature for 20 min. and an appropriate amount of diluted primary antibody working solution against target proteins (Cell Signaling, CDK4: #12790, 1:1000, Danvers, MA, USA; Proteintech, CTR1: #67221-1-Ig, 1:2000, Wuhan, China; Proteintech, Cytokeratin 17: 18502-1-AP, 1:100; Affinity Biosciences, ATP7A: #DF8506, 1:100, Shanghai, China; Affinity Biosciences, ATP7B: #AF0410, 1:100; Abcam, ASH1L: #ab234745, 1:1000, Cambridge, UK) was added dropwise. The sections were placed in a humidified box and incubated at 37°C for 1.5 h. After that, the sections were rinsed 3 times with PBS buffer (Gibco, #C10010500BT) for 5 min each time. Then, biotin-labeled secondary antibody (Thermo Fisher Scientific, Anti-Mouse: #31430, 1:500, Waltham, MA, USA; Thermo Fisher Scientific, Anti-Rabbit: #31460, 1:500, Waltham, MA, USA) working solution was added dropwise and incubated at 37°C for 30 min, followed by 3 rinses with PBS buffer (Gibco, #C10010500BT) for 5 min each time. Subsequently, horseradish peroxidase-labeled streptavidin working solution was added dropwise and incubated at 37°C for 30 min, with 3 more rinses with PBS buffer (Gibco, #C10010500BT) for 5 min each time. DAB (Solarbio, dilution ratio: 1:100, Catalog No.: SW1030-1kit, Beijing, China) chromogen was used for color development, and the staining time was controlled under a microscope, usually 3–10 min. After satisfactory color development, the reaction was terminated by thorough rinsing with tap water. Finally, the cell nuclei were counterstained with hematoxylin (Solarbio, Catalog No.: H8070-5, Beijing, China) for 2 min. After rinsing with running water, differentiation was performed with 1% hydrochloric acid ethanol for a few seconds, followed by washing with running water and placing in ammonia water for bluing. The sections were washed again with running water until the nuclei were clearly stained. The sections were sequentially immersed in 75% alcohol, 95% alcohol, 100% alcohol I, and 100% alcohol II for 3 min each. After natural drying, a drop of neutral gum was added to each slide for mounting. The slides were placed flat in a slide box, left in a fume hood overnight, and then stored in a slide cabinet at room temperature for subsequent observation of the expression and distribution of target proteins under a microscope (Olympus, BX53, Tokyo, Japan).

### 2.4 Method for Survival Analysis

Among the 197 renal tissue samples included in this study (all obtained from the Second Affiliated Hospital of Wenzhou Medical University, The clinical tissue samples were collected from 2018 to 2025), 15 were normal renal tissues, 153 were cisplatin-sensitive RCC tissues (Tumor group), and 29 were cisplatin-resistant RCC tissues (Tumor-DR group). The inclusion criteria were as follows: all samples were independently diagnosed by three experienced pathologists and confirmed as renal cell carcinoma (RCC) tissues or normal renal tissues; cisplatin-resistant RCC tissues (Tumor-DR group) were obtained from patients with disease progression or no response after at least 2 cycles of cisplatin-based chemotherapy, referring to the “Response Evaluation Criteria in Solid Tumors (RECIST 1.1)” and clinical practice; cisplatin-sensitive RCC tissues (Tumor group) were from patients who achieved partial response or complete response after cisplatin-based chemotherapy; normal renal tissues were derived from patients who underwent nephrectomy for non-tumor diseases (e.g., renal trauma, benign renal cyst) and confirmed to be tumor-free by pathological examination; all patients signed written informed consent for sample collection and research use. The exclusion criteria included patients with incomplete clinical and follow-up data, patients who received neoadjuvant radiotherapy or other targeted therapy before chemotherapy, samples with severe contamination, insufficient quantity or poor quality that could not meet the requirements of experiments such as immunohistochemical staining, and patients with concurrent other malignant tumors or severe systemic diseases (e.g., severe liver and kidney dysfunction, autoimmune diseases). Follow-up was conducted for 153 patients in the Tumor group and 29 patients in the Tumor-DR group. The follow-up strategy involved outpatient re-examinations (CT/MRI imaging and tumor marker detection) every 3 months within the first 2 years after treatment, and then every 6 months thereafter. Follow-up was initiated at the start of cisplatin chemotherapy for each patient, with the overall follow-up period covering from January 2018 to January 2025 (consistent with the core time range of sample collection). Regarding loss to follow-up and handling measures: no patients were lost to follow-up during the study period. To ensure the completeness and accuracy of follow-up data, the following measures were adopted: establishing a regular follow-up system, including actively reminding patients of outpatient re-examinations, conducting regular telephone follow-ups, and dynamically verifying electronic medical records; for patients unable to attend outpatient visits in person, conducting detailed telephone communications with the patients themselves or their immediate family members to confirm their health status, disease progression, and treatment situation; cross-validating all follow-up data with the hospital’s electronic medical record system and tumor registration database to eliminate conflicting information and ensure the authenticity and reliability of the data. The endpoint indicators were overall survival (OS, defined as the time from the initiation of cisplatin chemotherapy to death from any cause or the last follow-up) and progression-free survival (PFS, defined as the time from the initiation of cisplatin chemotherapy to disease progression or death). The Kaplan-Meier method was used to plot survival curves, and the log-rank test (two-sided test, with *p* < 0.05 considered statistically significant) was applied to compare survival differences between groups. Meanwhile, a Cox proportional hazards regression model was used to adjust for potential confounding factors to identify independent prognostic factors affecting the response to cisplatin treatment. The Schoenfeld residual test was used to verify the validity of the proportional hazards assumption. Complete Baseline Clinical Information and Follow-Up Data of Included RCC Patients (Table A1).

### 2.5 Cell Culture Experiment

A498 cells and RCC4 cells were used as the research objects. These cells were respectively inoculated into DMEM (Catalog No.: 11965092, Gibco) and RPMI1640 (Catalog No.: 27016021, Gibco) media containing 10% FBS (Catalog No.:10099158, Gibco) and 1% antibiotics (Catalog No.: 15140148, Gibco). All these media were provided by Gibco. The culture conditions were strictly controlled in a constant temperature and humidity environment of 37°C and 5% CO_2_ to simulate the optimal conditions for cell growth *in vivo*.

### 2.6 Growth Curve

A498 cells, RCC4 cells, and their cisplatin-resistant strains (A498-DR, RCC4-DR) in the logarithmic growth phase were selected and evenly seeded into 96-well plates at a density of 2 × 10^3^ cells per well, with 3 replicate wells set for each group. After seeding, the 96-well plate was placed in a constant temperature and humidity incubator at 37°C with 5% CO_2_ for culture. On days 0, 1, 2, 3, and 4 of culture, respectively, 10 μL of CCK-8 (MedChemExpress, HY-K0301) reagent was added to the wells corresponding to each time point, followed by continuous incubation for 2 h. Subsequently, a microplate reader (Thermo Fisher Scientific, Multiskan FC) was used to detect the absorbance value (OD value) of each well at a wavelength of 450 nm. A cell growth curve was plotted with the culture time as the abscissa and the OD value as the ordinate. The differences in proliferation rates among different cell strains (especially between drug-resistant strains and parental strains) were analyzed through the trend of the curve, so as to evaluate the cell growth ability and the impact of drug-resistant characteristics on proliferation, Proliferation rate = (OD_n_ − OD_0_)/OD_0_ × 100%, where n represents the culture days. All experiments were independently repeated three times.

### 2.7 Colony Formation Assay

A498 and RCC4 human renal cell carcinoma cell lines, along with their cisplatin-resistant sublines (A498-DR and RCC4-DR), were used as the research subjects. Cells in each group were seeded into 6-well plates at a density of 500 cells per well. Twenty-four hours after seeding, the cells were treated accordingly based on experimental requirements, and then cultured continuously for 6 days in a constant temperature and humidity incubator (Model: BB15, Thermo Fisher Scientific, Waltham, MA, USA) under conditions of 37°C and 5% CO_2_. After incubation, the medium in the wells was discarded, and the cells were washed twice with PBS buffer (Gibco, #C10010500BT). Then, 4% paraformaldehyde was added to fix the cells for 15 min. The fixative was discarded, followed by the addition of 0.1% crystal violet staining solution (Sigma-Aldrich, Cat. No.: HT90132, St. Louis, MO, USA) for 30 min of staining. After staining, excess staining solution was gently rinsed off with running water. Once the 6-well plates were air-dried, images of cells in each well were captured using a digital inverted microscope (Model: CKX53, Olympus Corporation, Tokyo, Japan). Colonies containing ≥ 50 cells per well were counted using ImageJ software (version 1.54f, Wayne Rasband, National Institutes of Health, Bethesda, MD, USA), and the colony formation rate was calculated (i.e., the ratio of the number of colonies in the experimental group to that in the control group × 100%). Three replicate wells were set up for each group of experiments, and the entire assay was independently repeated three times to ensure the reliability and reproducibility of the experimental results.

### 2.8 Wound Healing Assay

Logarithmic-phase A498, A498-DR, RCC4, and RCC4-DR cells were seeded into 6-well plates at a density of 5 × 10^5^ cells/well. Complete medium containing 10% FBS (Gibco, Cat. No.: 10099-141) was added, and the plates were incubated in a 37°C, 5% CO_2_ incubator until the cell confluency reached 90%–95%. Subsequently, the medium was replaced with serum-free medium (DMEM) (Catalog No.: 11965092, Gibco)/RPMI1640 (Catalog No.: 27016021, Gibco), and the cells were starved for 24 h to synchronize them in the G_0_/G_1_ phase, thereby eliminating the interference of cell proliferation on wound healing. A sterile 200 μL pipette tip was used to make a uniform scratch vertically at the center of the cell monolayer in each well (ensuring consistent scratch width across all wells, approximately 1 mm). The wells were gently washed 3 times with PBS buffer (Gibco, #C10010500BT) to remove detached cells generated by the scratching. According to the experimental groups, serum-free medium (DMEM) (Catalog No.: 11965092, Gibco)/RPMI1640 (Catalog No.: 27016021, Gibco) containing cisplatin at different concentrations (0.5 μg/mL for A498; 0.8 μg/mL for RCC4) was added, with 3 biological replicate wells set for each group. At 0 h, 6 h, 12 h, and 24 h after scratching, scratch images were captured using an inverted microscope (Olympus, BX53, Japan) at the same field of view (the coordinates at the bottom of the plate were marked to ensure consistent imaging positions at all time points).

### 2.9 In Vivo Experiments

Female BALB/c nude mice (n = 65, 4–6 weeks old, weighing 18–22 g) were purchased from Charles River Laboratories (Beijing, China). They were housed in a specific pathogen-free (SPF) environment (temperature: 22 ± 2°C, humidity: 50 ± 5%, 12-h light/dark cycle) with free access to food and water. After 1 week of adaptive feeding, three groups of A498-DR cells in the logarithmic growth phase were prepared: control group (untransfected), shNC group (transfected with negative control shRNA), and shCDK4 group (transfected with shCDK4-1 and shCDK4-2 interference sequences, respectively). All cells were resuspended in serum-free medium at a density of 5 × 10^6^ cells per mouse and inoculated subcutaneously into the right back of the nude mice. After inoculation, the mental state, diet, activity, and tumor growth of the nude mice were observed daily. The long diameter (L) and short diameter (W) of the tumors were measured with a vernier caliper every 3 days, and the tumor volume was calculated using the formula “Tumor volume (V) = 0.5 × L × W^2^” to plot the growth curve. When the tumor volume reached approximately 100 mm^3^, the nude mice were randomly divided into 9 groups (5 mice per group) using a random number table, and corresponding treatments were administered via intraperitoneal injection: control group (normal saline), shNC group, shCDK4-1 group, shCDK4-2 group, cisplatin (CIS, 5 mg/kg, #HY-17394, MedChemExpress) group, cisplatin (CIS, 5 mg/kg) + shCDK4 combination group, gemcitabine (GEM, 15 mg/kg, MedChemExpress, #HY-17026, Monmouth Junction, NJ, USA) group, shASH1L group, and gemcitabine (GEM, 15 mg/kg) + shASH1L combination group. The administration was performed twice a week for 3 consecutive weeks. During the experiment, the body weight of the nude mice was monitored daily to evaluate drug toxicity. If the nude mice showed signs of distress such as weight loss exceeding 10% or abnormal activity, humane endpoint measures were taken promptly. After the intervention, the nude mice were sacrificed by cervical dislocation. The tumor tissues were completely dissected, weighed, and then fixed in 4% paraformaldehyde for subsequent immunohistochemical detection of related proteins such as CDK4, ASH1L, and CTR1, so as to analyze the inhibitory effect of different treatments on tumor growth and the underlying molecular mechanisms. This animal experiment was approved by the Ethics Committee of the Second Affiliated Hospital of Wenzhou Medical University (Approval Number: 2025-KA-112-031), and all operations strictly followed animal welfare requirements and the ARRIVE Essential 10 guidelines to ensure transparent experimental design, reproducible data, and minimal animal suffering.

### 2.10 Transwell Assay Method

Target cells (A498, A498-DR, RCC4, RCC4-DR, siA498-DR and siRCC4-DR) in the logarithmic growth phase were harvested, resuspended in serum-free medium (DMEM, Catalog No.: 11965092, Gibco/RPMI1640, Catalog No.: 27016021, Gibco), and adjusted to a density of 5 × 10^4^ cells/mL. Transwell chambers with 8 μm pore-sized polycarbonate membranes (Corning Incorporated, #3422, Corning, NY, USA) were used. A total of 200 μL of the cell suspension was added to the upper chamber, and 600 μL of complete medium containing 10% FBS (Catalog No.:10099158, Gibco) and 1% hematoxylin (Catalog No.: H8070-5, Solarbio) was added to the lower chamber. For the drug-treated group, CIS was added to the lower chamber medium to a final concentration of 5 μg/mL, while no drug was added to the other groups. The chambers were incubated in a constant temperature and humidity incubator at 37°C with 5% CO_2_ for 24 h. After incubation, the chambers were removed, and the non-migrated cells in the upper chamber were gently wiped off with sterile cotton swabs. Subsequently, the chambers were placed in a petri dish containing 4% paraformaldehyde (Solarbio, #P1110, Beijing, China) and fixed at room temperature for 15 min. After fixation, the chambers were washed 3 times with PBS (Gibco, #C10010500BT, Waltham, MA, USA) buffer for 5 min each to remove residual fixative. Then, 0.1% crystal violet (#C8470, Solarbio) staining solution was added, and staining was performed at room temperature for 30 min. After staining, the chambers were gently rinsed with running water until excess staining solution was completely removed, and then air-dried naturally.

An inverted microscope (Olympus, IX73, Japan) was used to observe and capture images of migrated cells on the surface of the lower membrane of the chambers. Five random fields of view (upper, lower, left, right, and center) were photographed for each chamber. ImageJ software was used to count the migrated cells, with the counting standard being intact and clearly stained cells. The final results were expressed as “the mean number of cells ± standard deviation from 5 fields of view per group”. Each group was set with 3 replicate wells, and the experiment was independently repeated 3 times to ensure the reliability of the results.

### 2.11 Western Blot

A498, RCC4, A498-DR and RCC4-DR cells, as well as their transfected/intervened derivatives, were harvested and lysed in pre-chilled RIPA lysis buffer (Beyotime, Cat. No.: P0013, containing protease inhibitors, Shanghai, China) for 30 min on ice. The lysates were centrifuged at 12,075× *g* for 15 min at 4°C, and the supernatants were collected. The protein concentration was determined using a BCA Protein Assay Kit (Thermo Fisher Scientific, Cat. No.: 23225, Waltham, MA, USA). 5× SDS loading buffer (Solarbio, Cat. No.: PG112, Beijing, China) was added to the protein samples at a proportional volume, followed by protein denaturation via boiling at 100°C for 10 min. 10%–12% SDS-PAGE gels were prepared according to the molecular weights of target proteins, and 30 μg of protein was loaded into each well with a pre-stained protein Marker (Thermo Fisher Scientific, Cat. No.: 26616, 10–180 kDa, Waltham, MA, USA) as the reference. Electrophoresis was performed at a constant voltage of 80 V until the proteins entered the separating gel, then the voltage was adjusted to 120 V for an additional 90 min of electrophoresis. After electrophoresis, proteins were transferred from the gels to PVDF membranes (Beyotime, #BF7001, Shanghai, China) via the wet transfer method using transfer buffer (Beyotime, Cat. No.: P0021, 5×, Shanghai, China) at a constant current of 200 mA for 90 min. The PVDF membranes (Beyotime, #BF7001, Shanghai, China) were then blocked with 5% nonfat milk blocking buffer (BD Medical Devices Co., Ltd., Cat. No.: 232100, Shanghai, China) on a shaker at room temperature for 2 h. After discarding the blocking buffer, the membranes were washed three times with TBST buffer (10 min per wash), and then incubated with diluted primary antibodies at 4°C on a shaker overnight: CDK4 polyclonal antibody (Cell Signaling Technology, Cat. No.: 12790, Danvers, MA, USA), ASH1L polyclonal antibody (Abcam, Cat. No.: ab234745, Cambridge, UK), CTR1 polyclonal antibody (Abcam, Cat. No.: ab317432, Cambridge, UK), Vinculin monoclonal antibody (Sigma-Aldrich, Cat. No.: V9131, St. Louis, MO, USA), α-Tubulin (Cell Signaling Technology, Cat. No.: 2144, Danvers, MA, USA) and GAPDH monoclonal antibody (Proteintech Group, Inc., Cat. No.: 60004-1-Ig, Wuhan, China.), LAMP2 (Santa Cruz Biotechnology, Cat. No.: sc-18822, Dallas, TX, USA). Na^+^/K^+^-ATPase (Santa Cruz Biotechnology, Cat. No.: sc-514614, Dallas, TX, USA), FLAG (Flag) (Sigma-Aldrich, Cat. No.: F3040, St. Louis, MO, USA), HA (Hemagglutinin) (Abcam, Cat. No.: ab137838, Cambridge, UK), GST (Santa Cruz Biotechnology, Cat. No.: sc-138 (B-14), Dallas, TX, USA), All primary antibodies were diluted at a ratio of 1:1000, except for Vinculin and GAPDH antibodies which were diluted at 1:5000. On the following day, the membranes were washed three times with TBST buffer (10 min per wash), and incubated with HRP-conjugated goat anti-rabbit secondary antibody (Cell Signaling Technology, Cat. No.: 7074, Danvers, MA, USA) diluted at 1:5000 on a shaker at room temperature for 1 h. After another three washes with TBST buffer (10 min per wash), the membranes were incubated with the substrate from an ECL Chemiluminescence Kit (Thermo Fisher Scientific, Cat. No.: 32106, Waltham, MA, USA). Protein bands were captured using a chemiluminescence imaging system (Tanon Science & Technology Co., Ltd., Cat. No.: 5200Multi, Shanghai, China). Vertical electrophoresis (Bio-Rad Laboratories, Cat. No.: 1658001, Inc., Hercules, CA, USA) and electrotransfer (Bio-Rad Laboratories, Inc., Cat. No.: 1704150, Hercules, CA, USA) apparatuses were used for all electrophoresis and membrane transfer procedures throughout the experiment.

### 2.12 Molecular Docking Model Experiment

To conduct a molecular docking simulation study of ASH1L and CTR1, the authors selected the Autodock4 software (Scripps Research, San Diego, CA, USA). To accurately predict the interaction between ASH1L and CTR1, ASH1L (PDB:pdb_00008vlh) and CTR1 (PDB:pdb_00003p86) were downloaded from the Protein Data Bank (PDB, official website: https://www.rcsb.org/). The PyMoL software (Schrödinger, 2025-1, LLC, New York, NY, USA) was used to remove solvent molecules and ligands, completing the preparation of components for subsequent molecular docking. The PyMoL software was used to visually present the three-dimensional structure of the docking conformation with the lowest binding energy (ΔG). Meanwhile, the PLIP website (https://plip-tool.biotec.tu-dresden.de/plip-web/plip/index) was used to calculate the hydrogen bonding and hydrophobic interaction conditions.

### 2.13 Transcriptome Sequencing

During the experiment, A498 cells were first pretreated with Dapa reagent for 2 h and then treated with PA for 24 h. Subsequently, total mRNA was extracted using TRIzol reagent (Takara, #9109, Kyoto, Japan). The extracted mRNA samples were sent to Aksomics Inc. (Shanghai, China) for transcriptome sequencing and bioinformatics analysis (Project No.: H2407056). The sequencing was performed on an Illumina NovaSeq 6000 platform with paired-end 150 bp strategy. Differential expression analysis was conducted with Ballgown (v2.10.0) using criteria: Fold Change ≥ 1.5, *p*-value ≤ 0.05, and mean FPKM ≥ 0.5. Volcano plots and cluster analysis were generated using R packages ggplot2 (v3.4.4) and pheatmap (v1.0.12), respectively. All experimental data underwent strict quality control and were analyzed and provided by the technical team of Aksomics Inc. (Shanghai, China) to ensure the accuracy and reliability of the results. The results of transcriptome sequencing have been uploaded to the Zenodo database (https://doi.org/10.5281/zenodo.17934004).

### 2.14 Cell Knockdown (siRNA)

In the cell knockdown (siRNA) experiment, A498 and RCC4 cell lines were selected. One day before transfection, cells were seeded into 6-well plates at a density of 2 × 10^5^ cells per well and cultured until the cell confluency reached 50%–60%. Before the experiment, the cells were washed twice with PBS (Gibco, #C10010500BT) pre-equilibrated to room temperature, followed by replacement with serum-free medium. The transfection mixture was prepared in the following proportions: per well, 80 μL of Opti-MEM™ medium (Thermo Fisher Scientific, #51985091, Waltham, MA, USA), 4 μL of Lipofectamine 2000 transfection reagent (Thermo Fisher Scientific, #11668019, Waltham, MA, USA), and 2 μL each of specific siRNAs (siRNA-1 and siRNA-2, GenePharma, Suzhou, China) were added, with a final concentration of 50 nM for each siRNA and a total volume of the transfection mixture of 88 μL. Meanwhile, a negative control si-NC group (transfected with non-targeting negative control siRNA) and a blank control mock group (only transfection reagent and Opti-MEM™ medium added, no siRNA) were set up. After incubating the prepared transfection mixture at room temperature for 20 min, it was evenly added dropwise to each well of the 6-well plate, which was then placed in a 37°C, 5% CO_2_ incubator for 6 h. Subsequently, the medium was replaced with complete medium containing 10% fetal bovine serum, and the cells were cultured for an additional 48 h. Transfection efficiency was verified by qPCR (detecting the mRNA expression level of the target gene) and Western blot (detecting the protein expression level of the target gene). After confirming that the knockdown efficiency was ≥70%, the cells were collected for subsequent experiments, and the primer information is detailed in Table A2.

### 2.15 CUT&Tag Sequencing Experimental Method

Collect A498-DR cells in the logarithmic growth phase, and perform cell fixation and magnetic bead binding using Magna ChIP™ Protein A + G Magnetic Beads (Merck Millipore Co., Ltd., Cat. No.: 16-663, Burlington, MA, USA). Resuspend the cells in lysis buffer prepared with Pierce™ Protease Inhibitor Cocktail (Thermo Fisher Scientific Inc., Cat. No.: 88666, Waltham, MA, USA). After lysing on ice for 30 min, add the diluted rabbit anti-human CDK4 polyclonal antibody (Cat. No.: 12790, Cell Signaling Technology) at a dilution ratio of 1:1000, and incubate overnight on a shaker at 4°C. The next day, add goat anti-rabbit IgG secondary antibody (Jackson ImmunoResearch Laboratories Inc., Cat. No.: 111-035-003, West Grove, PA, USA) at a dilution ratio of 1:5000, incubate at room temperature for 1 h, and then add the Protein A-Tn5 transposase complex (Thermo Fisher Scientific, Cat. No.: E55150, Waltham, MA, USA) to incubate at room temperature for 1 h to complete the cleavage and tagging of target sites. Purify and recover the cleaved DNA fragments using the QIAquick PCR Purification Kit (Qiagen, Cat. No.: 28104, Hilden, Germany), and construct the library with the NEBNext^®^ Ultra™ II DNA Library Prep Kit (New England Biolabs, Cat. No.: E7645L, Ipswich, MA, USA). After the constructed library passes quality inspection with the Agilent 2100 Bioanalyzer (Agilent Technologies, Cat. No.: G2939BA, Santa Clara, CA, USA), perform high-throughput sequencing on the Illumina NovaSeq 6000 sequencing platform (Cat. No.: 20024906, Illumina, San Diego, CA, USA) with a paired-end 150 bp sequencing strategy. The raw sequencing data are aligned using Bowtie2 software (v2.4.5) against the hg38 reference genome, and peak calling is performed with MACS2 software (v2.2.7.1) to identify the genomic binding sites of the CDK4 protein.

### 2.16 ChIP Experimental Methodology

Collect A498-DR cells in the logarithmic growth phase and fix them with 1% paraformaldehyde solution (Sigma-Aldrich, Cat. No.: P6148, St. Louis, MO, USA) at room temperature for 10 min. Add glycine solution (Sigma-Aldrich, Cat. No.: G8898, St. Louis, MO, USA) to terminate the fixation. After washing the cells three times with PBS buffer (Gibco, #C10010500BT), resuspend them in RIPA lysis buffer (Beyotime, Cat. No.: P0013, Shanghai, China) containing Pierce™ Protease Inhibitor Cocktail (Thermo Fisher Scientific, Cat. No.: 88666, Monmouth Junction, NJ, USA) and Phosphatase Inhibitor Cocktail (Beyotime, Cat. No.: P0044, Shanghai, China), followed by lysis on ice for 30 min. Use an ultrasonic disruptor (Sonics & Materials, Inc., Cat. No.: VCX130, Newtown, CT, USA) to shear chromatin into 200–500 bp fragments. Centrifuge at 12,075× *g* for 15 min at 4°C to collect the supernatant, and verify the fragment size of part of the sample by agarose gel electrophoresis.

Take 50 μL of the chromatin supernatant as the Input control. Add rabbit anti-human CDK4 polyclonal antibody (Cat. No.: 12790, Cell Signaling Technology) to the remaining sample at a dilution ratio of 1:100, and incubate overnight on a shaker at 4°C. Meanwhile, set up normal rabbit IgG control (Cell Signaling Technology, Cat. No.: 2729, USA) to exclude non-specific binding. The next day, add Magna ChIP™ Protein A + G Magnetic Beads (Cat. No.: 16-663, Merck Millipore Co., Ltd.) to the reaction system. After incubating at 4°C for 4 h, wash the beads sequentially four times (5 min each time) with low-salt wash buffer, high-salt wash buffer, LiCl wash buffer, and TE buffer (Merck Millipore Co., Ltd., Cat. No.: 17-295, MA, USA). Add ChIP elution buffer (Merck Millipore Co., Ltd., Cat. No.: 17-296, Darmstadt, Germany) to the washed beads and incubate at room temperature for 15 min to elute the immune complexes. Collect the eluate and mix it with the Input sample, then add Proteinase K solution (New England Biolabs Inc, Cat. No.: P8107S, USA) and incubate at 65°C for 4 h to reverse cross-linking. Purify and recover the DNA fragments using the QIAquick PCR Purification Kit (Qiagen, Cat. No.: 28104, Venlo, The Netherlands). Perform qPCR detection using SYBR^®^ Fast qPCR Mix (Takara Bio Inc., Cat. No.: RR430S, Kusatsu, Japan) and the CFX96 Real-Time System (Bio-Rad Laboratories Inc., Cat. No.: 1855196, USA).

### 2.17 RNA Extraction and qPCR

Total RNA was extracted from the cultured cell lines using the RNApure Tissue & Cell Kit (Jiangsu Kangwei Century Biotechnology Co., Ltd., #CW0584S, Taizhou, China), RNA purity was detected with a Nanodrop 2000 spectrophotometer (Thermo Fisher Scientific, #ND-2000, Waltham, MA, USA) to ensure the A260/A280 ratio ranged between 1.8 and 2.0; RNA concentration was measured using a Qubit 4 fluorometer (Thermo Fisher Scientific, #Q33238, Waltham, MA, USA) to guarantee a concentration of ≥50 ng/μL per sample; RNA integrity was verified by 1% agarose gel electrophoresis, ensuring clear bands of 28S and 18S rRNA with a brightness ratio close to 2:1. Subsequently, reverse transcription was performed using the HiFiScript cDNA Synthesis Kit (Jiangsu Kangwei Century Biotechnology Co., Ltd., #CW0584S, Suzhou, China) in a 20 μL reaction system containing 1 μg of total RNA, 4 μL of 5× HiFiScript Buffer (Jiangsu Kangwei Century Biotechnology Co., Ltd., CW0584S), 1 μL of HiFiScript Enzyme Mix (Jiangsu Kangwei Century Biotechnology Co., Ltd., CW0584S), 1 μL of Oligo (dT) 18 Primer, 1 μL of Random Hexamer Primer (Jiangsu Kangwei Century Biotechnology Co., Ltd., CW0584S), and RNase-free water. The reaction conditions were: incubation at 25°C for 10 min (primer binding), 50°C for 30 min (reverse transcription), and 85°C for 5 min (enzyme inactivation). Quantitative PCR (qPCR) analysis was conducted using the reverse-transcribed cDNA as a template, with SYBR^®^ Fast qPCR Mix (TaKaRa, #RR430, Japan) and the CFX96 Real-Time System (Bio-Rad Laboratories Inc.#185-5096, Hercules, CA, USA). The 20 μL qPCR reaction system included 10 μL of 2× SYBR^®^ Fast qPCR Mix (TaKaRa, #RR430, Japan), 0.4 μL of forward primer (10 μM), 0.4 μL of reverse primer (10 μM), 2 μL of cDNA template (1:10 dilution), and 7.2 μL of enzyme-free water. The thermal cycling conditions were: pre-denaturation at 95°C for 30 s, followed by 40 cycles of denaturation at 95°C for 5 s and annealing/extension at 60°C for 30 s. After cycling, melt curve analysis was performed (from 65°C to 95°C, increasing by 0.5°C every 5 s) to verify primer specificity. GAPDH (Proteintech Group, Inc., Cat. No.: 60004-1-Ig) was used as the internal reference gene to normalize the mRNA expression levels of target genes, and the 2^−ΔΔCt^ method was employed to calculate relative expression levels. PCR amplification efficiency was verified by the standard curve method, ensuring all primers had an amplification efficiency between 90% and 110%. Primer information is detailed in Table A3 (GenePharma, Suzhou, China).

### 2.18 Mass Spectrometry Analysis of ASH1L Peptides

The immunoprecipitated ASH1L protein was separated by 10% SDS-PAGE (gel preparation kit: Beyotime, Cat. No.: P0012A, Shanghai, China) and stained with Coomassie Brilliant Blue R-250 (Solarbio, Cat. No.: C0121, Beijing, China). The target protein band was excised, destained with 50% acetonitrile (ACN; Thermo Fisher Scientific, Cat. No.: A955-4, Waltham, MA, USA) in 25 mM ammonium bicarbonate (Sigma-Aldrich, Cat. No.: A6141, St. Louis, MO, USA), and digested with sequencing-grade modified trypsin (Promega, Cat. No.: V5111, Madison, WI, USA) at a ratio of 1:50 (enzyme:protein, w/w) at 37°C for 16 h. The digestion was terminated by adding 1% trifluoroacetic acid (TFA; Thermo Fisher Scientific, Cat. No.: 28904, Waltham, MA, USA), and the peptides were extracted sequentially with 50% ACN/0.1% TFA and 100% ACN/0.1% TFA (ACN and TFA from Thermo Fisher Scientific, Cat. No.: A955-4 and 28904, respectively, Waltham, MA, USA). Desalted peptides were analyzed by liquid chromatography-tandem mass spectrometry (LC-MS/MS) using a Q Exactive HF-X mass spectrometer (Thermo Fisher Scientific, #IQLAAEGAAPFALGMAZX, Waltham, MA, USA) coupled to an Easy-nLC 1200 ultra-high-performance liquid chromatography system (Thermo Fisher Scientific, #LC120, Waltham, MA, USA). Peptides were loaded onto a trap column (Acclaim PepMap 100, 75 μm × 2 cm, C18, 3 μm; Thermo Fisher Scientific, Cat. No.: 164564, Waltham, MA, USA) and separated on an analytical column (Acclaim PepMap RSLC, 75 μm × 15 cm, C18, 2 μm; Thermo Fisher Scientific, Cat. No.: 164943, Waltham, MA, USA) with a linear gradient of 2–35% ACN (Thermo Fisher Scientific, Cat. No.: A955-4, Waltham, MA, USA) in 0.1% formic acid (Thermo Fisher Scientific, Cat. No.: 28905, Waltham, MA, USA) over 60 min at a flow rate of 300 nL/min. The mass spectrometer was operated in data-dependent acquisition (DDA) mode: Full MS scan: 350–1500 m/z, resolution 120,000, AGC target 3e6, maximum injection time 50 ms. Top 20 most intense precursor ions, resolution 30,000, AGC target 1e5, maximum injection time 20 ms, isolation window 1.6 m/z, normalized collision energy (NCE) 28%.

### 2.19 Co-IP

One day before transfection, 293T cells were seeded into 6-well plates at a density of 5 × 10^5^ cells per well and cultured until the cell confluency reached 70%-80% for co-transfection. The transfection system per well consisted of 1 μg of Flag-CTR1 expression plasmid (GenePharma, Cat. No.: GP-PL001, Suzhou, China), 1 μg of HA-ASH1L expression plasmid (GenePharma, Cat. No.: GP-PL002, Suzhou, China), 3 μL of Lipofectamine 3000 transfection reagent (Thermo Fisher Scientific, Cat. No.: L3000015, Waltham, MA, USA), and 100 μL of Opti-MEM™ medium (Thermo Fisher Scientific, Cat. No.: 31985070, Waltham, MA, USA), with a total volume of 105 μL. After incubating at room temperature for 20 min, the mixture was evenly added dropwise to the cells, which were then cultured in a 37°C, 5% CO_2_ incubator for 48 h. After transfection, the cells were collected and lysed on ice for 30 min with RIPA lysis buffer (Beyotime, #P0013B, Shanghai, China) containing 1% protease inhibitor (PMSF, Solarbio, Cat. No.: P8340, Beijing, China). Total protein extract was obtained by centrifugation at 12,075× *g* at 4°C for 15 min. Meanwhile, Input control (20 μL of total protein extract directly used for subsequent detection), IgG negative control (2 μL of non-specific IgG antibody diluted 1:500; Cell Signaling Technology, Cat. No.: 2729, Danvers, MA, USA), and beads-only control (Protein A/G agarose beads without antibody) were set up. A total of 500 μg of total protein extract was taken, and 2 μL of Flag tag-specific antibody (diluted 1:500, Cell Signaling Technology, Cat. No.: 2368, Danvers, MA, USA) or HA tag-specific antibody (diluted 1:500, Cat. Abcam, No.: ab9110, Cambridge, UK) was added, followed by gentle shaking incubation at 4°C overnight. The next day, 50 μL of Protein A/G agarose beads (50% slurry; Thermo Fisher Scientific, Cat. No.: 20421, Waltham, MA, USA) was added, and incubation was continued at 4°C for 3 h. After incubation, the precipitate was collected by centrifugation at 3000× *g* at 4°C for 5 min, washed 4 times with pre-cooled RIPA lysis buffer (5 min each time), and the supernatant was completely aspirated after the last centrifugation. The precipitated complex was collected for subsequent Western blot detection and analysis.

### 2.20 A498-DR Cell Direct Co-IP System

A498-DR cells in the logarithmic growth phase were collected and lysed on ice for 30 min using RIPA lysis buffer (Beyotime, Cat. No.: P0013B) containing 1% protease inhibitor (PMSF, Solarbio, Cat. No.: P8340, Beijing, China). Total protein was extracted by centrifugation at 12,075× *g* at 4°C for 15 min, and the protein concentration was determined using a BCA Protein Quantification Kit (Thermo Fisher Scientific, Cat. No.: 23225, Waltham, MA, USA). A total of 800 μg of total protein extract was used for immunoprecipitation: 1.6 μL of ASH1L-specific primary antibody (dilution ratio 1:500, Cell Signaling Technology, USA, Cat. No.: 14994, Danvers, MA, USA) or 1.6 μL of CTR1-specific primary antibody (dilution ratio 1:500, Abcam, Cat. No.: ab185839, Cambridge, UK) was added respectively, followed by gentle shaking incubation at 4°C overnight. The next day, 60 μL of Protein A/G agarose beads (50% slurry, Thermo Fisher Scientific, Cat. No.: 20421, Waltham, MA, USA) was added, and incubation was continued at 4°C for 3 h. After incubation, the precipitate was collected by centrifugation at 3000× *g* at 4°C for 5 min, washed 4 times with pre-cooled RIPA lysis buffer (5 min each time, Beyotime, Cat. No.: P0013B). After purifying the complex, it was subjected to Western blot detection together with the products from the aforementioned 293T cell system.

### 2.21 GST-Pull down

First, GST-tagged fusion target protein expression plasmids (GST-ASH1L and GST-CTR1, GenePharma, Cat. Nos.: GP-PL003 and GP-PL004, respectively, Suzhou, China) were constructed and transformed into the prokaryotic expression strain BL21 (DE3) (TransGen Biotech, Cat. No.: CD601-02, Beijing, China). When the OD600 value of the bacterial culture reached 0.6–0.8, isopropyl-β-D-thiogalactoside (IPTG, Solarbio, Cat. No.: I8150, Beijing, China) was added to a final concentration of 0.5 mM, and protein expression was induced by shaking at 37°C for 4 h. After induction, the bacterial cells were collected and resuspended in bacterial lysis buffer (Beyotime, Cat. No.: P0013B, Shanghai, China), supplemented with 1× protease inhibitor cocktail (Beyotime, Cat. No.: SC0263-10mM, Shanghai, China) Following ultrasonic disruption, the lysate was centrifuged at 12,075× *g* at 4°C for 15 min to obtain the supernatant. Then, 50 μL of glutathione agarose beads (50% slurry, Thermo Fisher Scientific, Cat. No.: 16100, Waltham, MA, USA) was added to the supernatant, and the mixture was gently shaken and incubated at 4°C for 2 h. After washing 3 times with pre-cooled wash buffer (centrifuged at 3000× *g* at 4°C for 5 min each time), purified “agarose bead-GST fusion protein” complexes were obtained, with both GST-ASH1L and GST-CTR1 at a concentration of 0.5 μg/μL. Meanwhile, His-tagged candidate proteins (His-CTR1 and His-ASH1L, GenePharma, Cat. Nos.: GP-PL005 and GP-PL006, respectively, Suzhou, China) were prepared using an *in vitro* transcription-translation system (Promega, Cat. No.: L1170, Madison, WI, USA) and adjusted to a concentration of 0.4 μg/μL. The experiment included three essential controls: GST alone pull-down control (glutathione agarose beads + GST protein only), His protein alone control (His-tagged protein + glutathione agarose beads only), and Input control (10 μL of His-tagged candidate protein directly used for Western blot detection). Next, 20 μL of the “agarose bead-GST fusion protein” complex was mixed with 30 μL of His-tagged candidate protein, and the volume was made up to 200 μL with wash buffer. The mixture was gently shaken and incubated at 4°C for 4 h to ensure sufficient interaction. After incubation, the precipitate was collected by centrifugation at 3000× *g* at 4°C for 5 min, washed 4 times with pre-cooled wash buffer (5 min each time), and the supernatant was completely aspirated after the final centrifugation. Then, 20 μL of 2× SDS loading buffer (Beyotime, Cat. No.: P0015, Shanghai, China) was added, and the sample was denatured by boiling at 100°C for 5 min. After SDS-PAGE electrophoresis, the samples were transferred to a PVDF membrane (Beyotime, #BF7001). The membrane was blocked with 5% non-fat milk powder (Becton, Dickinson and Company, Cat. No.: 232100, Franklin Lakes, New Jersey) at room temperature for 1 h, then incubated with a His-tag-specific primary antibody (dilution ratio 1:1000, Cell Signaling Technology, Cat. No.: 2365, Danvers, MA, USA) at 4°C overnight. On the next day, the membrane was washed 3 times with TBST buffer (Beyotime, #P0023, Shanghai, China), followed by incubation with an HRP-conjugated goat anti-rabbit secondary antibody (dilution ratio 1:5000, Thermo Fisher Scientific, Cat. No.: 31460, Waltham, MA, USA) at room temperature for 1 h. After another 3 washes with TBST buffer, the membrane was developed using an ECL chemiluminescence kit (Thermo Fisher Scientific, Cat. No.: 32106, Waltham, MA, USA), and images were captured with the ImageQuant LAS 4000 imaging system (GE Healthcare, USA). The loading volume for each sample was 15 μL. The direct protein-protein interaction between ASH1L and CTR1 was confirmed by detecting the presence of the His-tagged candidate protein.

### 2.22 Fluorescence Co-Localization

Target cells in good growth condition (e.g., A498-DR cells) were seeded into 24-well plates containing cell climbing slices at a density of 2 × 10^4^ cells per well, and cultured in a 37°C, 5% CO_2_ incubator for 24 h until the cell confluency reached 60%–70%; the control group was cultured routinely. Subsequently, the cells were fixed with 4% paraformaldehyde (Beyotime, Cat. No.: P0098, Shanghai, China) at room temperature for 15 min, and washed 3 times with PBS buffer (Gibco, #C10010500BT) (5 min each time). A PBS buffer (Gibco, #C10010500BT) containing 0.1% Triton X-100 was added to permeabilize the cell membrane at room temperature for 10 min. After washing 3 times with PBS (Gibco, #C10010500BT), a blocking solution containing 5% bovine serum albumin (BSA, Solarbio, Cat. No.: A8010) was added for blocking at room temperature for 1 h to reduce non-specific binding. After blocking, a diluted mixture of specific primary antibodies (anti-ASH1L primary antibody: Cell Signaling Technology, USA, Cat. No.: 14994, dilution ratio 1:500; anti-CTR1 primary antibody: Abcam, UK, Cat. No.: ab185839, dilution ratio 1:500) was added, or a mixture of anti-CTR1 primary antibody with the cell membrane marker Wheat Germ Agglutinin (WGA), (dilution ratio 1:200, Thermo Fisher Scientific, Cat. No.: W11261, Waltham, MA, USA) and the lysosome marker LysoTracker Red (Thermo Fisher Scientific, Cat. No.: L7528, Waltham, MA, USA, working concentration 75 nM), followed by incubation at 4°C overnight. On the next day, the cells were washed 3 times with PBS (5 min each time, Gibco, #C10010500BT), and the corresponding fluorochrome-labeled secondary antibodies (Alexa Fluor 488-conjugated goat anti-rabbit secondary antibody: Thermo Fisher Scientific, Cat. No.: A11008, dilution ratio 1:1000; Alexa Fluor 594-conjugated goat anti-mouse secondary antibody: Thermo Fisher Scientific, Cat. No.: A11005, dilution ratio 1:1000) were added, and incubated at room temperature in the dark for 1 h. After washing 3 times with PBS (Gibco, #C10010500BT), DAPI staining solution (Solarbio, Cat. No.: C0060, dilution ratio 1:1000) was used for staining at room temperature in the dark for 5 min, followed by another 3 washes with PBS (Gibco, #C10010500BT). Finally, the slices were mounted with anti-fluorescence quenching mounting medium (Beyotime, Cat. No.: P0126, Shanghai, China). Images were observed and captured using an LSM 880 laser confocal microscope (Carl Zeiss, #LSM 880, Jena, Germany). The overlap of fluorescent signals from different target proteins was analyzed to clarify the colocalization relationship between proteins and the distribution characteristics of CTR1 (Abcam, Cat. No.: ab317432) in subcellular structures such as the cell membrane and lysosomes.

### 2.23 Surface Plasmon Resonance (SPR) Experimental Methods

The recombinant CTR1 protein was immobilized on the surface of a CM5 sensor chip (Biacore-compatible, GE Healthcare, Cat. No.: BR-1005-30, Marlborough, MA, USA) via amine coupling to maintain its native conformation. The critical parameters of the coupling reaction were strictly controlled as follows: the immobilization concentration of recombinant CTR1 (Abcam, Cat. No.: ab317432) was 50 μg/mL; the coupling reaction was performed in 10 mM sodium acetate buffer (pH 4.5, Sigma-Aldrich, Cat. No.: S8625, St. Louis, MO, USA) using N-hydroxysuccinimide (NHS, Sigma-Aldrich, Cat. No.: 130672, St. Louis, MO, USA) and 1-ethyl-3-(3-dimethylaminopropyl) carbodiimide hydrochloride (EDC, Sigma-Aldrich, Cat. No.: E7750, St. Louis, MO, USA) as activation reagents, with an activation time of 7 min and a coupling reaction time of 15 min; after coupling, 1 M ethanolamine hydrochloride (pH 8.5, Sigma-Aldrich, Cat. No.: E1759, St. Louis, MO, USA) was used to block unreacted active sites for 7 min. Subsequently, ASH1L protein at gradient concentrations was used as the mobile phase, and continuously flowed over the chip surface at a constant flow rate of 30 μL/min in PBS buffer (Gibco, #C10010500BT) at 25°C (temperature fluctuation controlled within ±0.1°C). The association time was set to 120 s, and the dissociation time was 300 s. Resonance signal changes at different concentrations were recorded in real-time to generate sensorgrams. The sensorgrams were subjected to data fitting and analysis using the software accompanying the Biacore T200 instrument (Version 3.0, GE Healthcare, Uppsala, Sweden). The equilibrium dissociation constant (Kd value) was calculated using the 1:1 Langmuir binding model to verify the specificity and affinity of the interaction between the two proteins. A blank control channel (immobilized with 50 μg/mL bovine serum albumin, BSA, Sigma-Aldrich, Cat. No.: A7906) was set up during the experiment to subtract non-specific binding signals. Each concentration gradient was repeated three times as technical replicates, and the entire experiment was independently repeated three times as biological replicates to ensure the reliability and reproducibility of the data.

### 2.24 Construction of CTR1, CDK4 and ASH1L Overexpression Plasmids

For the overexpression of CTR1, CDK4, and ASH1L, the pcDNA3.1(+) eukaryotic expression vector (Invitrogen™, Thermo Fisher Scientific, Cat. No.: V79020, Waltham, MA, USA) was used. The full-length CDS sequences of human CTR1 (NCBI reference sequence: NM_001859.3), CDK4 (NCBI reference sequence: NM_000075.4), and ASH1L (NCBI reference sequence: NM_001130835.2) were amplified separately via PCR using the human renal cortex cDNA library (BioVector NTCC Inc., Cat. No.: BC118596, Beijing, China) as the template and PrimeSTAR^®^ Max DNA Polymerase (Takara Bio Inc., Cat. No.: R045A, Kusatsu, Japan). Both the amplified products and the vector were subjected to double digestion with EcoR I (New England Biolabs Inc., Cat. No.: R0101S, Ipswich, MA, USA) and Xho I (New England Biolabs Inc., Cat. No.: R0146S, Ipswich, MA, USA), followed by ligation using T4 DNA ligase (New England Biolabs Inc., Cat. No.: M0202S, Ipswich, MA, USA) to generate the recombinant plasmids pcDNA3.1-CTR1, pcDNA3.1-CDK4, and pcDNA3.1-ASH1L. The ligation products were then transformed into DH5α competent cells (TransGen Biotech, Cat. No.: CD201-02, Beijing, China), and positive clones were screened with ampicillin (Sigma-Aldrich, Cat. No.: A9518, St. Louis, MO, USA). For transfection, the verified recombinant plasmids were mixed with Lipofectamine 3000 transfection reagent (Thermo Fisher Scientific, Cat. No.: L3000015, Waltham, MA, USA) at an appropriate ratio, incubated at room temperature for 20 min, and subsequently added to A498, RCC4, A498-DR, or RCC4-DR cells at 60% confluence. Six hours after transfection, the medium was replaced with DMEM medium (Gibco, Cat. No.: 11965092, Waltham, MA, USA) supplemented with 10% fetal bovine serum (Gibco, Cat. No.: 10099141, Waltham, MA, USA). The overexpression efficiency was verified by Western blot 48 h post-transfection.

### 2.25 Luciferase Reporter Gene Assay

A luciferase reporter gene vector containing the wild-type ASH1L promoter (ASH1L WT, GenePharma, Cat. No.: GP-PL007, Suzhou, China) was constructed. For the three potential CDK4 binding sites on the ASH1L promoter (Binding site 1: TGTGGGGAGAAG, Binding site 2: TGTCAACCCACT, Binding site 3: AGTGGCGTGATC), single-point mutant vectors (MUT1, MUT2, MUT3, GenePharma, Cat. Nos.: GP-PL008, GP-PL009, GP-PL010, respectively, Suzhou, China) were constructed using a site-directed mutagenesis kit (Agilent, Cat. No.: 200518, Santa Clara, CA, USA). The empty vector pGL4.10 without the promoter fragment (Promega, Cat. No.: E6651, Madison, WI, USA) was used as the negative control (pCMV-Vector). The CDK4 overexpression vector (pCMV-CDK4, GenePharma, Cat. No.: GP-PL011, Suzhou, China) was constructed by cloning the full-length coding region of CDK4 into the pCMV-HA vector (Clontech, Cat. No.: 6083-1, CA, USA), and the empty vector pCMV-HA (Clontech, USA, Cat. No.: 6083-1, CA, USA) served as the control for the overexpression experiment. All vectors were verified by sequencing (Sangon Biotech Co., Ltd., Shanghai, China). One day before transfection, A498-DR cells were seeded into 24-well plates at a density of 2 × 10^4^ cells per well and cultured in DMEM medium containing 10% fetal bovine serum (Gibco, Cat. No.: 11965118) in a 37°C, 5% CO_2_ incubator until the cell confluency reached 60%–70%. The transfection system was prepared in the following proportions: per well, 0.8 μg of the reporter gene vector (ASH1L WT/MUT1/MUT2/MUT3/pCMV-Vector), 0.4 μg of the CDK4 overexpression vector (pCMV-CDK4) or empty vector control (pCMV-HA), 0.04 μg of the internal reference vector pRL-TK (containing the Renilla luciferase gene, Promega, Cat. No.: E2241, Madison, USA) to correct transfection efficiency, 2 μL of Lipofectamine 3000 transfection reagent (Thermo Fisher Scientific, Cat. No.: L3000015, Waltham, MA, USA), and Opti-MEM™ medium (Thermo Fisher Scientific, Cat. No.: 31985070 Waltham, MA, USA) to make up the total volume to 100 μL. The mixture was incubated at room temperature for 20 min to form a transfection complex. The complex was evenly added dropwise to each well of the 24-well plate, and the cells were cultured for an additional 48 h before collection. Detection was performed using a dual-luciferase assay kit (Promega, Cat. No.: E1910, Madison, USA) strictly following the kit instructions: after discarding the medium, 100 μL of Passive Lysis Buffer (Promega, Cat. No.: E1941, Madison, USA) was added to each well, and the cells were lysed by shaking at room temperature for 15 min. Twenty microliters of the cell lysate was transferred to a 96-well white luminescent plate, 100 μL of Luciferase Assay Reagent II was added to detect Firefly luciferase activity, followed by 100 μL of Stop & Glo^®^ Reagent to detect Renilla luciferase activity. Readings were taken using a GloMax^®^ 20/20 luminometer (Promega, Cat. No.: E5311, Madison, USA), and the relative luciferase activity was expressed as the ratio of “Firefly luciferase activity/Renilla luciferase activity”. Each experiment was set with 3 technical replicates and independently repeated 3 times to ensure biological reproducibility. Data were statistically analyzed using GraphPad Prism 8.0 software: one-way analysis of variance (ANOVA) was used for comparisons among multiple groups, and Dunnett’s multiple comparison test was used for pairwise comparisons between groups. A *p*-value < 0.05 was considered statistically significant. By comparing the differences in luciferase activity among different vector groups, the effect of CDK4 on ASH1L promoter activity and the role of specific binding sites were analyzed.

### 2.26 Cycloheximide (CHX)-Mediated Protein Synthesis Inhibition Assay

A498-DR cells transfected with siNC or siASH1L were treated with 50 μg/mL CHX (MedChemExpress, Cat. #HY-12320, Monmouth Junction, NJ, USA) to block *de novo* protein synthesis, and cells were harvested at 0, 6, 12, and 24 h post-treatment; total proteins were extracted using RIPA lysis buffer (Beyotime, Cat. No.: P0013, Shanghai, China) supplemented with protease inhibitors, protein concentration was determined by BCA assay (Thermo Fisher Scientific, Cat. No.: 23225, Waltham, MA, USA), and equal amounts of proteins were subjected to SDS-PAGE electrophoresis (Bio-Rad Laboratories Inc., Cat. No.: 1658001, Hercules, CA, USA) followed by Western blot.

### 2.27 Gray Value Statistical Method

Gray value analysis of protein bands was performed using ImageJ software (Version 1.54f, Wayne Rasband, National Institutes of Health, Bethesda, MD, USA). First, band images acquired by the Western blot chemiluminescence imaging system (Tanon Science & Technology Co., Ltd., Cat. No.: 5200Multi, Shanghai, China) were imported. Using the built-in “Analyze-Gels” function of the software, the band regions of target proteins (CTR1, ASH1L) and internal reference proteins (α-Tubulin, GAPDH) were manually selected. After automatic background gray value subtraction, the Integrated Optical Density (IOD) of each band was obtained. The ratio of the IOD value of the target protein to that of the corresponding internal reference protein was used as the relative expression level of the protein. Each sample was set with 3 biological replicates and 3 technical replicates, and the final data were expressed as “mean ± standard deviation (Mean ± SD)”. Statistical analysis was performed using GraphPad Prism 10 software (GraphPad, San Diego, CA, USA).

### 2.28 Statistics

In this study, all experimental data are presented as the mean ± standard error of the mean (SEM). To ensure the reliability and reproducibility of the results, each experiment was independently repeated three to six times. For the comparison of two groups of data, we used the two-sample Student’s *t*-test; for the analysis of multiple groups of data, one-way analysis of variance (ANOVA) was used, and further evaluation of the differences between groups was carried out through Dunnett’s multiple comparison test. Data analysis was performed using GraphPad Prism software (Version 8.0, GraphPad, San Diego, CA, USA) to determine the statistical significance between the treatment group and the control group. The statistical significance levels were set at *p < 0.05, **p < 0.01, and ***p < 0.001, indicating statistically significant, highly significant, and extremely significant differences, respectively.

## 3 Results

### 3.1 High Expression of CDK4 Is Associated with RCC Onset, Cisplatin Resistance, and Poor Patient Prognosis

**Figure 1:**
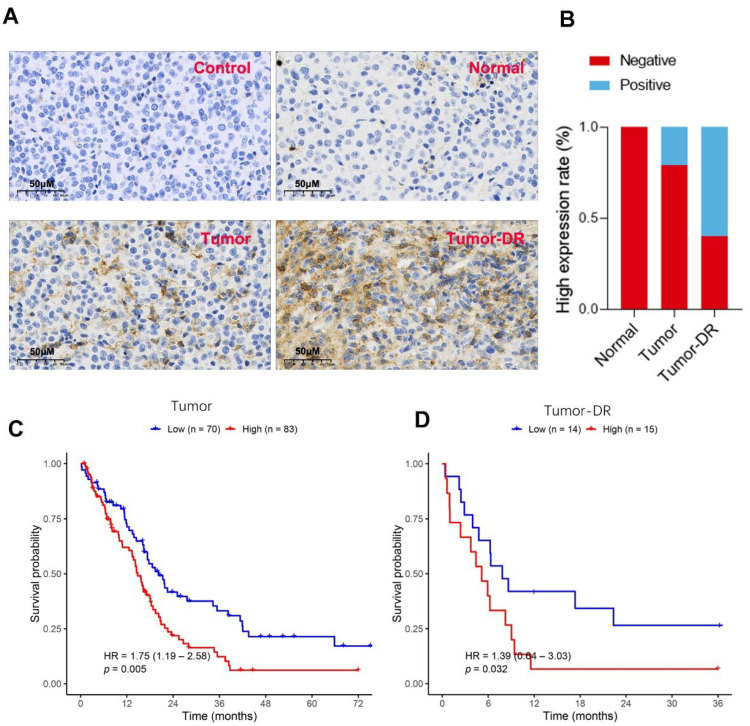
**Cyclin-dependent kinase 4 (CDK4) staining results.** (**A**): CDK4 expression in Control, Normal, Tumor, and Tumor-DR tissues. (**B**): High expression rate of CDK4 in Normal, Tumor, and Tumor-DR tissues. (**C**): Survival curve of the Tumor group based on CDK4 expression. (**D**): Survival curve of Tumor-DR group based on CDK4 expression.

Results of immunohistochemical staining (Fig. 1A) showed that there was almost no positive expression of CDK4 in the Control group, and only weak positive expression in the Normal group. In contrast, obvious positive CDK4 staining was observed in the Tumor group and Tumor-DR group, with significantly stronger positive expression in the Tumor-DR group. Subsequently, statistics on the high expression rate of CDK4 in specimens from different groups (Fig. 1B) indicated that the high expression rate was extremely low in the Normal group, increased in the Tumor group, and further significantly elevated in the Tumor-DR group. This suggests that high CDK4 expression is closely associated with RCC onset and drug resistance. To clarify the relationship between CDK4 expression and patient prognosis, survival analysis was performed. The results showed that: in the Tumor group (Fig. 1C), the survival probability of patients with high CDK4 expression (High) was significantly lower than that of patients with low CDK4 expression (Low), with a hazard ratio (HR) of 1.75 and *p* = 0.005; in the Tumor-DR group (Fig. 1D), the survival probability of patients with high CDK4 expression was also significantly lower than that of patients with low expression, with an HR of 1.39 and *p* = 0.032. In conclusion, high CDK4 expression is not only associated with RCC onset and drug resistance, but also significantly correlated with poor prognosis in patients with RCC and cisplatin-resistant RCC.

### 3.2 Biological Behavior Differences between Cisplatin-Resistant RCC Cells and Parental Cells, and Characteristics of CDK4 Expression

**Figure 2:**
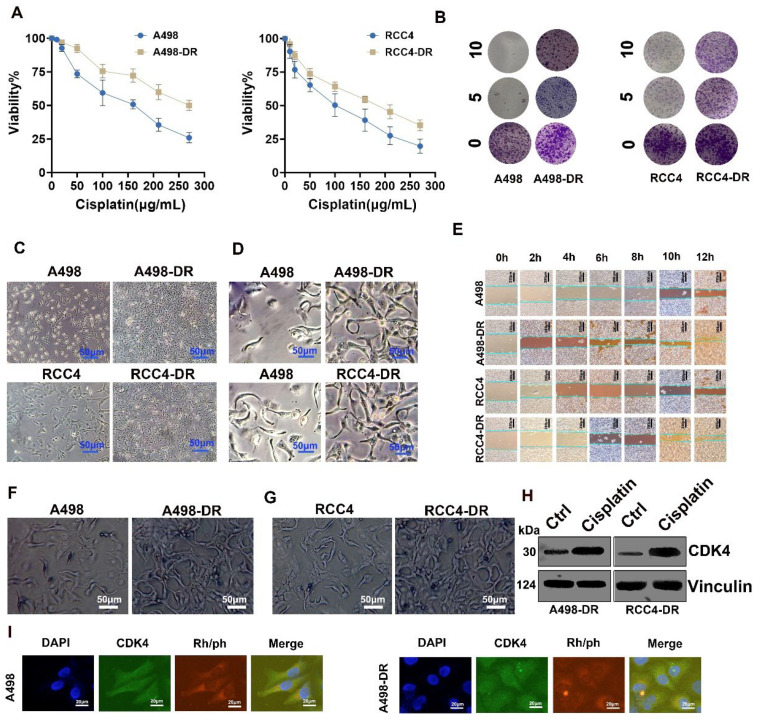
**Biological Characteristics of Cisplatin-Resistant renal cell carcinoma (RCC) Cells and Parental Cells.** (**A**,**B**): Effects of cisplatin on the viability of A498, A498-DR, RCC4, and RCC4-DR cells; (**C**,**D**): Observation of cell morphology, bar: 50 μm; (**E**): Wound healing assay to detect cell migration; bar: 50 μm; (**F**,**G**): Representative results of the Transwell migration assay: Comparison between the normal group and the drug-resistant group. (**H**,**I**): Western blot and Immunofluorescence staining (bar: 20 μm) to detect CDK4 expression. The treatment time for cisplatin was consistently 24 h. Ctrl: Control.

Results of the cell viability assay (Fig. 2A) showed that with the increase in cisplatin concentration, the viability of A498, A498-DR, RCC4, and RCC4-DR cells all showed a downward trend. However, compared with the parental A498 and RCC4 cells, the cisplatin-resistant A498-DR and RCC4-DR cells (drug-resistant strains) exhibited stronger tolerance to cisplatin, with higher viability at the same cisplatin concentration.

**Figure A1:**
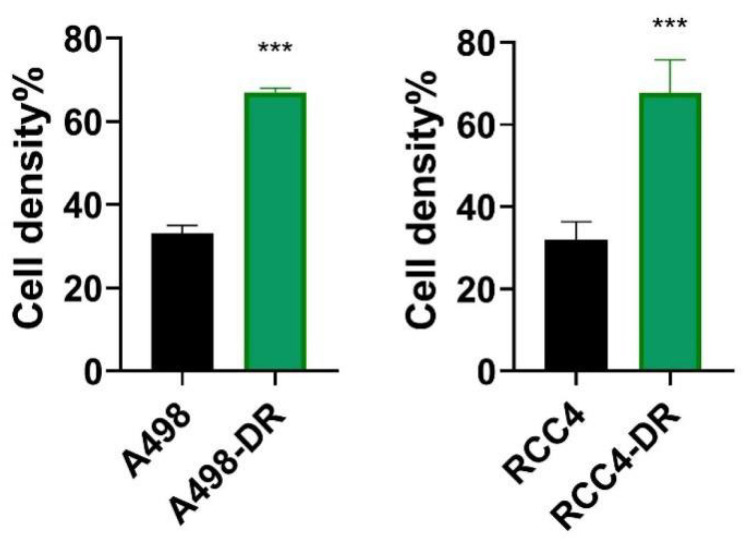
Transwell assay was performed to detect the percentage of transmembrane cell density in parental A498/RCC4 cells and drug-treated (DR) groups; black bars represent parental groups, and green bars represent DR groups; data are presented as cell density percentage (Cell density%), and inter-group differences were analyzed by *t*-test, RCC: Renal Cell Carcinoma. ***p < 0.001.

**Figure A2:**
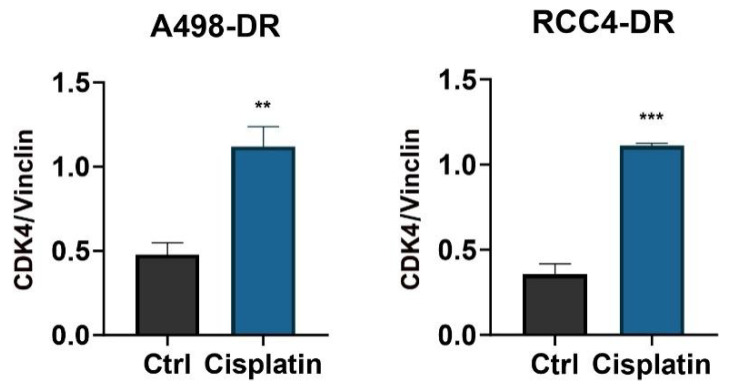
Effect of cisplatin treatment on the relative expression levels of cyclin-dependent kinase 4 (CDK4)/Vinclin protein (normalized band gray value results) in A498-DR and RCC4-DR cells. Ctrl: control group; Cisplatin: cisplatin treatment group. Data are presented as mean ± standard deviation, analyzed by *t*-test, n = 3; **p < 0.01, ***p < 0.001.

The colony formation assay (Fig. 2B) demonstrated that the colony-forming ability of A498-DR and RCC4-DR cells was significantly stronger than that of the parental A498 and RCC4 cells, suggesting that drug-resistant cells have stronger proliferative capacity. Observation of cell morphology (Fig. 2C,D) revealed morphological differences between A498-DR/RCC4-DR cells and their parental counterparts, with the drug-resistant cells showing more aggressive phenotypic characteristics. Results of the wound healing assay (Fig. 2E) showed that the migration ability of A498-DR and RCC4-DR cells was significantly higher than that of the parental cells, with faster wound healing rates at different time points. Compared with normal A498 and RCC4 cells, A498DR and RCC4DR cells exhibited a stronger ability to migrate through the Transwell chambers (Fig. 2F,G and Fig. A1). In addition, results of Western blot analysis indicated that the expression of CDK4 in A498-DR and RCC4-DR cells increased significantly after cisplatin treatment (Fig. 2H and Fig. A2). Immunofluorescence results indicated that the expression level of CDK4 in A498DR cells was significantly higher than that in A498 cells (Fig. 2I). In summary, there are differences in biological behaviors (such as proliferation and migration) between cisplatin-resistant RCC cells and parental RCC cells, and these differences may be related to the expression of CDK4 and its regulation by cisplatin.

### 3.3 CDK4 Promotes Proliferation and Migration of RCC and Cisplatin-Resistant RCC Cells and Reduces Cisplatin Sensitivity

**Figure 3:**
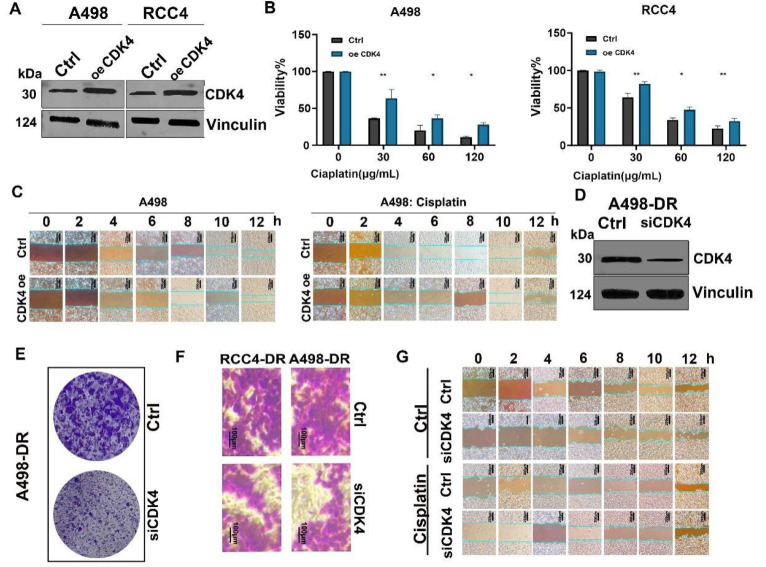
**Effects of CDK4 on RCC A498, RCC4, and Their Drug-Resistant Strains.** (**A**): Western blot to detect CDK4 overexpression; (**B**): Changes in cell viability; (**C**): Wound healing assay (Left panel: Proliferation status of A498 cells (0–12 h) after CDK4 overexpression under regular culture conditions; Right panel: Proliferation inhibition of CDK4-overexpressing A498 cells (0–12 h) under cisplatin treatment.); (**D**): Verification of CDK4 knockout in A498-DR cells; (**E**): Colony formation of A498-DR cells; (**F**): Representative image of Transwell results, bar:100 μm; (**G**): Wound healing assay of A498-DR cells (including cisplatin-treated group). (Two-way ANOVA), Mean ± SD (Standard Deviation), n = 3, *p < 0.05; **p < 0.01.

**Figure A3:**
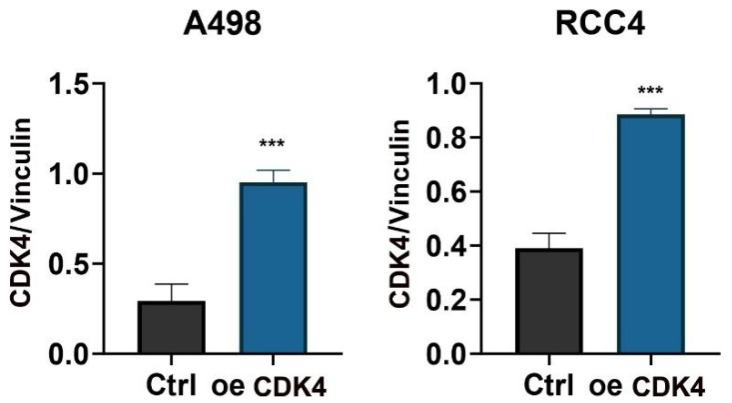
Effect of CDK4 overexpression on the relative expression levels of CDK4/Vinclin protein (normalized band gray value results) in A498 and RCC4 cells. Ctrl: control group; oe CDK4: CDK4 overexpression group. Data are presented as mean ± standard deviation, analyzed by *t*-test (n = 3); ***p < 0.001.

**Figure A4:**
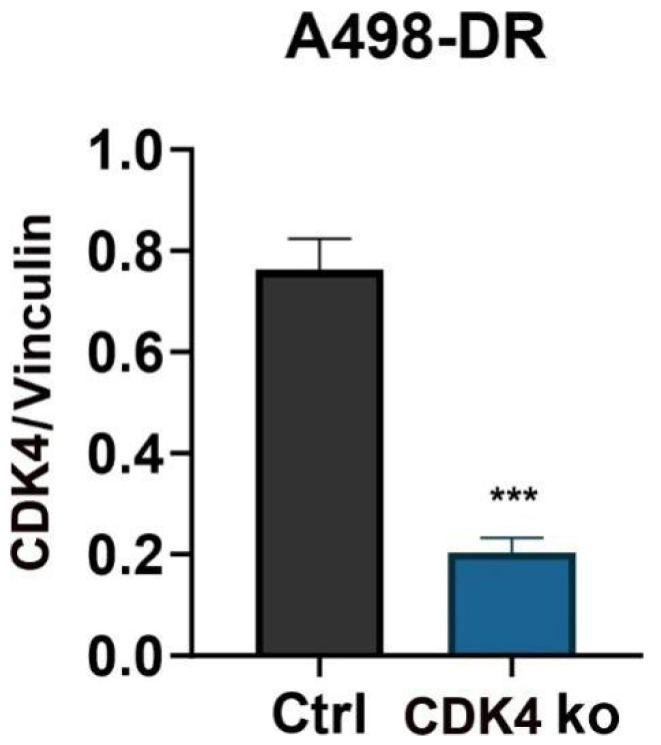
Effect of CDK4 knockout on the relative expression levels of CDK4/Vinclin protein (normalized band gray value results) in A498-DR cells. Ctrl: control group; CDK4 ko: CDK4 knockout group. Data are presented as mean ± standard deviation, analyzed by *t*-test (n = 3); ***p < 0.001.

Western blot results (Fig. 3A and Fig. A3) showed that in A498 and RCC4 cells, after CDK4 overexpression (oe CDK4), the protein level of CDK4 increased significantly, while the expression of internal reference GAPDH remained stable, indicating reliable overexpression efficiency. Cell viability assay results (Fig. 3B) demonstrated that compared with the control group (Ctrl), the viability of A498 and RCC4 cells with CDK4 overexpression decreased more slowly under cisplatin treatment, suggesting that CDK4 overexpression can reduce cell sensitivity to cisplatin. Results of the wound healing assay (Fig. 3C) showed that the wound healing rate of A498 cells with CDK4 overexpression was faster than that of non-overexpressing cells in both the control group and cisplatin-treated group, reflecting the promoting effect of CDK4 on cell migration ability. In A498-DR cells, after CDK4 knockout (ko CDK4) (Fig. 3D and Fig. A4); Western blot verified the knockout efficiency, with a significant decrease in CDK4 protein level and stable GAPDH expression), the colony formation assay (Fig. 3E) showed that the colony-forming ability of CDK4-knockout cells was significantly weaker than that of the control group; The sphere migration assay showed that knocking out CDK4 in A498-DR cells led to a decrease in cell migration ability, and the migration range of cells in the CDK4 knockout group was smaller than that in the control group (Fig. 3F). Further wound healing assay (Fig. 3G) indicated that the migration ability of CDK4-knockout A498-DR cells decreased significantly in both the control group and cisplatin-treated group. In conclusion, CDK4 can promote the proliferation and migration of RCC and cisplatin-resistant RCC cells, and reduce cell sensitivity to cisplatin, playing an important role in the occurrence, development, and drug resistance of RCC.

### 3.4 CDK4 Knockdown Inhibits Tumorigenicity and Enhances Cisplatin Sensitivity of Resistant RCC Cells

**Figure 4:**
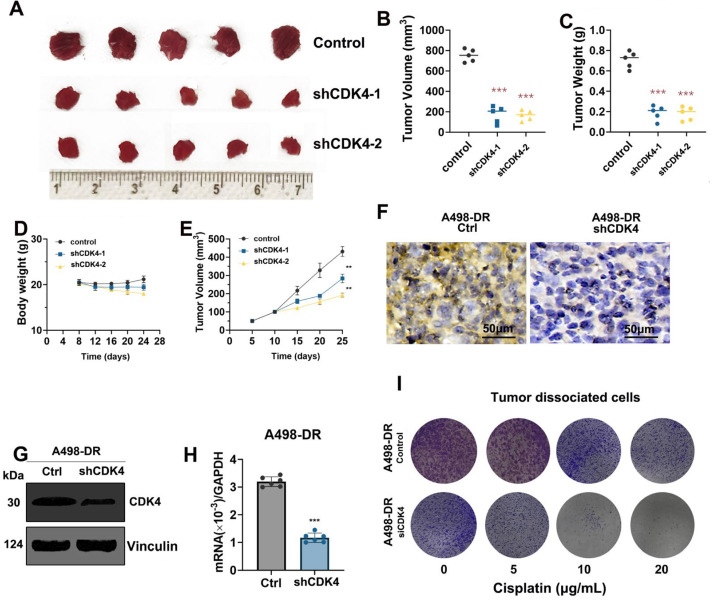
**RCC Xenografts in Nude Mice and Related Detection.** (**A**): Photographs of mice and tumors; (**B**–**E**): Changes in tumor volume, tumor weight, and nude mouse body weight; (**F**): Staining of tumor tissues, bar: 50 μm; (**G**): Western blot to detect CDK4; (**H**): Quantitative real-time polymerase chain reaction (qPCR) to detect CDK4; (**I**): Cisplatin treatment of tumor-dissociated cells; Mean ± SD, **p < 0.01; ***p < 0.001. SD, standard deviation.

**Figure A5:**
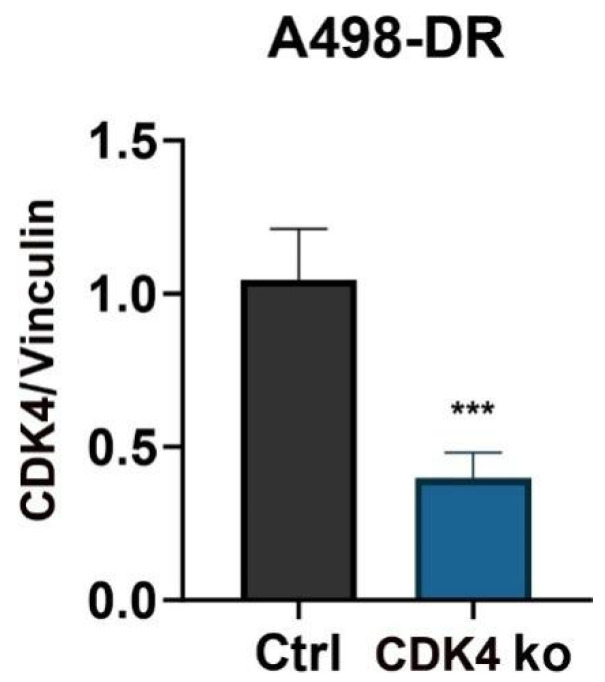
Effect of CDK4 knockout on the relative expression levels of CDK4/Vinclin protein (normalized band gray value results) in A498-DR cells. Ctrl: control group; CDK4 ko: CDK4 knockout group. Data are presented as mean ± standard deviation, analyzed by *t*-test (n = 3); ***p < 0.001.

Tumorigenesis experiments in nude mice (Fig. 4A) showed that tumors in the Control group were relatively large, while those in the shCDK4-1 and shCDK4-2 groups were significantly smaller. Further quantitative analysis (Fig. 4B,C) indicated that the tumor volume and weight in the shCDK4-1 and shCDK4-2 groups were significantly lower than those in the Control group. At the same time, there was no significant difference in the body weight of nude mice among all groups (Fig. 4D), suggesting that CDK4 knockdown had little impact on the overall health of nude mice. The time-dependent curve of tumor volume (Fig. 4E) further confirmed the inhibitory effect of CDK4 knockdown on tumor growth. In experiments related to A498-DR cells, histological staining (Fig. 4F) revealed differences between the CDK4 knockout (CDK4 ko) group and the control group (Ctrl). Western blot (Fig. 4G and Fig. A5) verified that the expression of CDK4 protein in the CDK4 ko group was significantly reduced. The tumor volume curve (Fig. 4H) reflected the inhibitory effect of CDK4 ko on tumor growth. In the tumor-dissociated cell experiment (Fig. 4I), with the increase of cisplatin concentration, cells in the CDK4 ko group were more significantly inhibited, indicating that CDK4 knockout enhanced the sensitivity of A498-DR cells to cisplatin.

### 3.5 CDK4 Regulates Gene Expression in RCC Cells by Modulating ASH1L Promoter Activity

**Figure 5:**
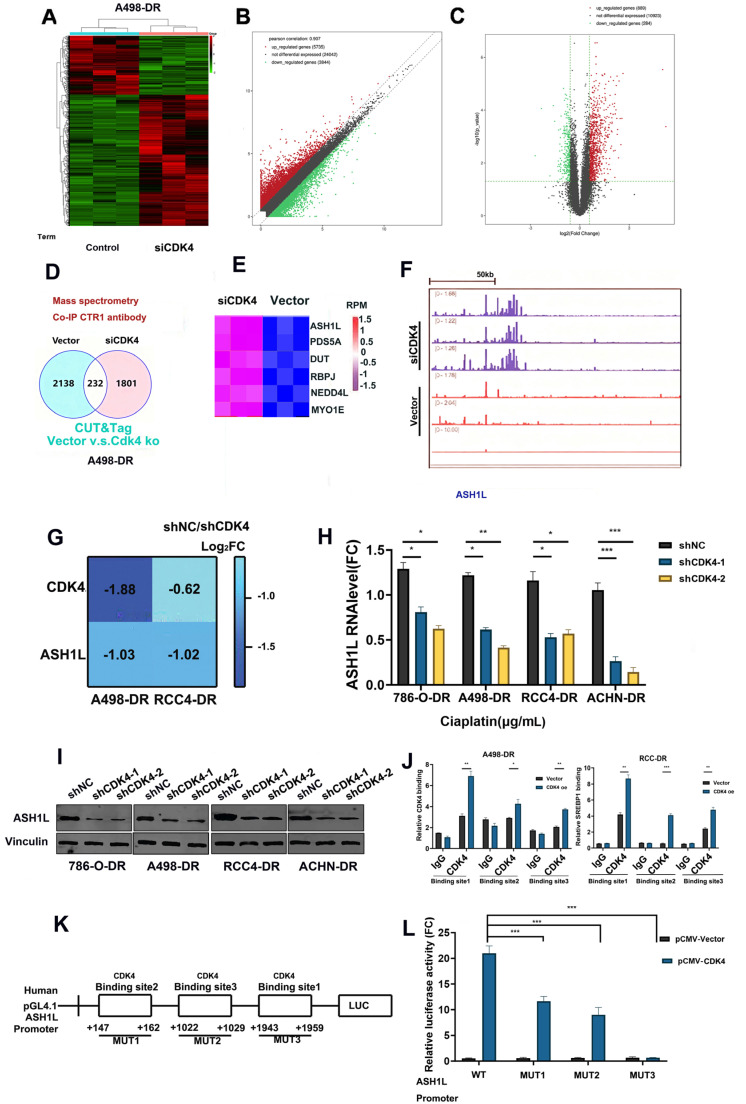
**CDK4-Related Omics and Functional Verification.** (**A**): Heatmap of differentially expressed genes; (**B**,**C**): Scatter plots of gene expression; (**D**): Combined analysis of mass spectrometry and CUT&Tag; (**E**): Gene expression after CDK4 knockout; (**F**): Sequencing of the ASH1L region; (**G**): Expression of CDK4 and ASH1-Like Histone Lysine Methyltransferase (ASH1L) in RCC cell lines; (**H**): ASH1L mRNA level; (**I**): ASH1L protein expression; (**J**): Chromatin immunoprecipitation (ChIP)-qPCR to verify binding; (**K**): Binding sites between the ASH1L promoter and CDK4; (**L**): Dual-luciferase reporter gene assay. (One-way ANOVA). *p < 0.05; **p < 0.01; ***p < 0.001.

To further explore the downstream molecular regulatory mechanism of CDK4, a series of experiments was conducted. First, gene chip technology (Fig. 5A) was used to analyze the gene expression profiles of the control group and CDK4 knockout (CDK4 Ko) group in A498-DR cells. Scatter plots (Fig. 5B) were used to visually present the overall differences in gene expression between the two groups, while volcano plots (Fig. 5C) were used to further screen for significantly differentially expressed genes. Subsequently, combined with co-immunoprecipitation (Co-IP) technology and CUT&Tag experiments (Fig. 5D), molecules that might interact with CDK4 were screened and verified, and several potential CDK4-interacting proteins were identified. Cluster analysis of differentially expressed genes (Fig. 5E) clearly showed differences in the expression patterns of different genes between the control group and the CDK4 Ko group. Using chromosome region binding maps (Fig. 5F), the specific binding region of CDK4 to the target gene (ASH1L) on chromosomes was identified.

**Figure A6:**
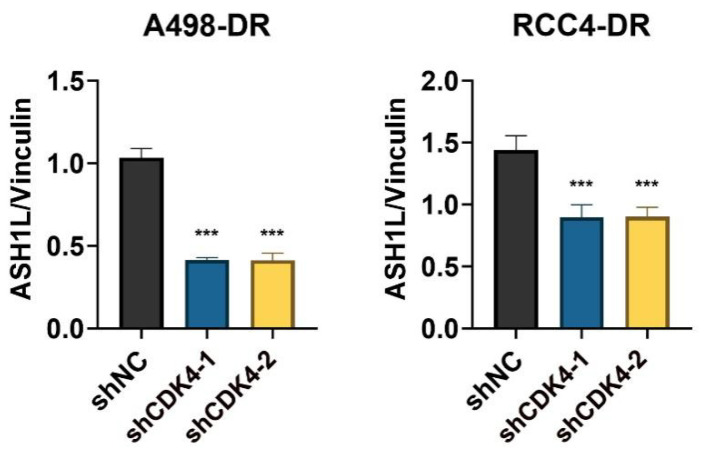
Effect of CDK4 knockdown on the relative expression levels of ASH1L/Vinculin protein (normalized band gray value results) in A498-DR and RCC4-DR cells. shNC: negative control group; shCDK4-1, shCDK4-2: CDK4 knockdown groups (two interference sequences). Data are presented as mean ± standard deviation, analyzed by *t*-test (n = 3); ***p < 0.001.

In RCC cell lines (including A498-DR, RCC4-DR), after CDK4 knockdown (shCDK4-1 and shCDK4-2 groups), qPCR detection (Fig. 5G,H) showed that the mRNA level of ASH1L was significantly reduced. Western blot experiments (Fig. 5I and Fig. A6) also confirmed that the protein expression of ASH1L decreased significantly after CDK4 knockdown, while the expression of the internal reference protein Vinculin remained stable, indicating that CDK4 knockdown could downregulate ASH1L expression.

Results of further ChIP experiments (Fig. 5J) showed that CDK4 could directly bind to the promoter region of the ASH1L gene, and the binding status differed among different binding sites. To verify the functionality of CDK4 binding sites, luciferase reporter gene vectors containing different mutant binding sites (MUT1, MUT2, MUT3 corresponding to Binding site 1, 2, 3) of the ASH1L promoter were constructed (Fig. 5K), and their relative luciferase activity was detected (Fig. 5L). The results showed that compared with the control group (pCMV-Vector), overexpression of CDK4 (pCMV-CDK4) significantly increased the luciferase activity of the wild-type (ASH1L WT) ASH1L promoter. However, when the binding sites on the promoter were mutated (MUT1, MUT2, MUT3), the regulatory effect of CDK4 on promoter activity was significantly weakened, especially for MUT2 and MUT3. This further confirms that CDK4 regulates the activity of the ASH1L promoter through specific binding sites.

### 3.6 ASH1L Affects CTR1 Protein Levels by Regulating Its Lysosomal Pathway-Mediated Degradation

**Figure 6:**
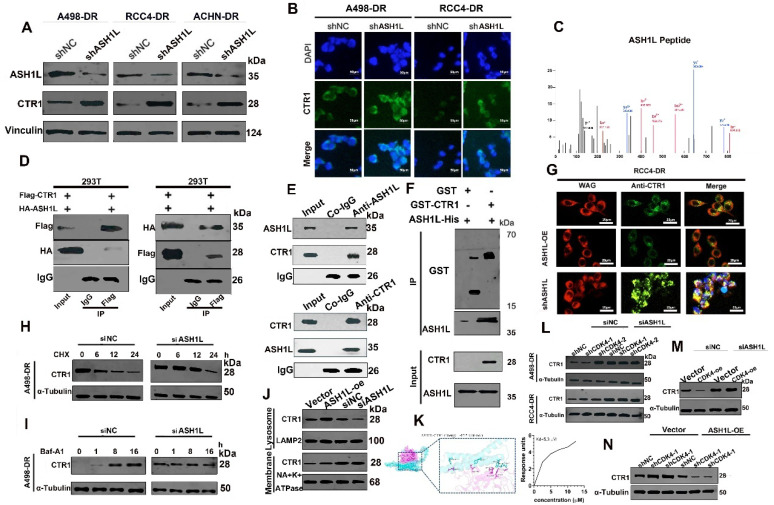
**Interaction and Function of ASH1L and copper transporter 1 (CTR1).** (**A**): Western blot to detect CTR1 expression after ASH1L knockdown; (**B**): Immunofluorescence staining of CTR1; (**C**): Mass spectrometry of ASH1L peptides; (**D**,**E**): Co-immunoprecipitation to verify the binding between ASH1L and CTR1; (**F**): GST pull-down experiments; (**G**): Immunofluorescence co-localization; (**H**,**I**): Western blot was performed to detect the protein expression level of CTR1, with α-Tubulin serving as the loading control; (**J**): CTR1 expression in membrane proteins; (**K**): Binding model of ASH1L and CTR1; (**L**–**N**): Western blot was performed to detect the protein expression level of CTR1, with α-Tubulin serving as the loading control.

**Figure A7:**
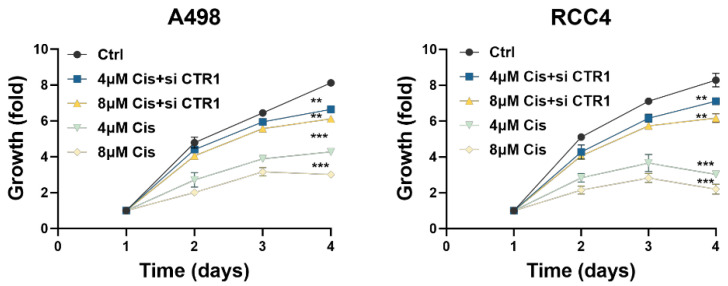
A498 and RCC4 cells were treated with control (Ctrl), cisplatin alone (4/8 μM Cis), or cisplatin plus copper transporter 1 (CTR1) silencing (Cis + si CTR1), and the changes in growth fold were recorded from day 0 to 4. Data are presented as mean ± standard deviation, with **p < 0.01 and ***p < 0.001 (vs. the Cis group at the same concentration, n = 5).

**Figure A8:**
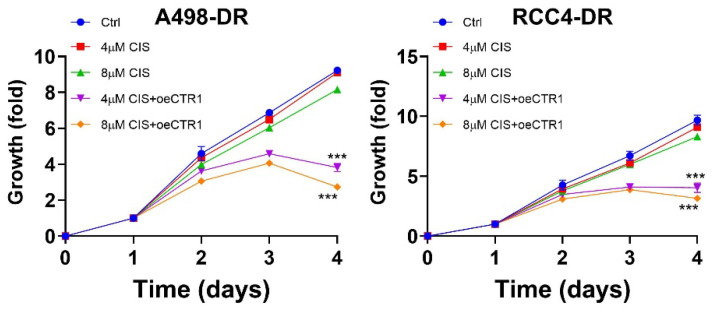
A498-DR and RCC4-DR cells were treated with control (Ctrl), cisplatin alone (4/8 μM CIS), or cisplatin plus CTR1 overexpression (CIS + oeCTR1), and the changes in growth fold were recorded from day 0 to 4. Data are presented as mean ± standard deviation, with ***p < 0.001 (vs. the CIS group at the same concentration, n = 5).

**Figure A9:**
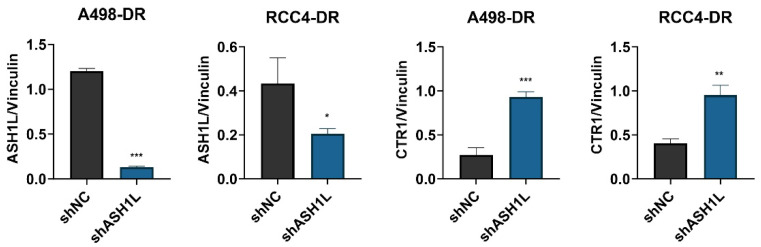
Effect of ASH1L knockdown on the relative expression levels of ASH1L/Vinculin and CTR1/Vinculin protein (normalized band gray value results) in A498-DR and RCC4-DR cells. shNC: negative control group; shASH1L: ASH1L knockdown group. Data are presented as mean ± standard deviation, analyzed by *t*-test (n = 3); *p < 0.05, **p < 0.01, ***p < 0.001.

In this study, the critical regulatory role of CTR1 in cisplatin response was further validated through *in vitro* proliferation assays using A498, RCC4, A498-DR, and RCC4-DR cells. In the parental cell model, treatment with cisplatin alone (4 μM/8 μM Cis) significantly inhibited cell proliferation; however, after CTR1 downregulation via siRNA, the growth fold of cells in the cisplatin-treated group was notably elevated compared to the cisplatin-only group (Fig. A7), indicating that CTR1 downregulation can attenuate the growth-inhibitory effect of cisplatin on parental cells and enhance cellular resistance to cisplatin. In the drug-resistant cell model, the proliferation-inhibitory effect of cisplatin alone (4 μM/8 μM CIS) was weak; in contrast, after combining cisplatin treatment with CTR1 overexpression (oeCTR1), the cell growth fold was significantly reduced (Fig. A8), which further reversely validates the negative regulatory relationship between CTR1 expression level and cisplatin response. To further explore the interaction between ASH1L and CTR1 and the regulatory mechanism of ASH1L on CTR1, a series of experiments was conducted. First, after ASH1L knockdown in A498-DR and RCC4-DR cells, Western blot analysis (Fig. 6A and Fig. A9) showed that the CTR1 protein level was significantly upregulated, indicating that ASH1L exerts a negative regulatory effect on CTR1.

**Figure A10:**
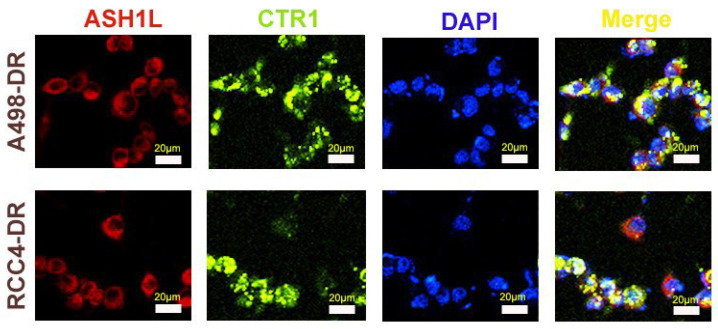
Fluorescence co-localization analysis of ASH1L (red) and CTR1 (green) in A498-DR and RCC4-DR cells. DAPI (blue) labels cell nuclei, and Merge is the merged image. Bar:20 μm.

Subsequently, immunofluorescence experiments (Fig. 6B) revealed that after ASH1L knockdown, the fluorescence signal intensity of CTR1 was significantly enhanced, and ASH1L and CTR1 colocalized (Fig. A10). This preliminarily suggested a potential interaction between the two proteins. To further confirm this interaction, mass spectrometry analysis of ASH1L peptides was performed (Fig. 6C), providing a molecular basis for subsequent studies.

Next, the results of co-immunoprecipitation (Co-IP) experiments (Fig. 6D,E) showed that the interaction between ASH1L and CTR1 could be detected both in 293T cells co-transfected with Flag-CTR1 and HA-ASH1L (followed by IP) and in A498-DR cell lysates (directly subjected to Co-IP), confirming the binding relationship between the two proteins. GST pull-down experiments (Fig. 6F) further demonstrated a direct protein-protein interaction between ASH1L and CTR1.

**Figure A11:**
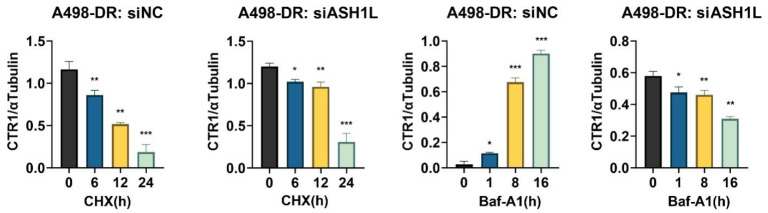
Effect of ASH1-Like Histone Lysine Methyltransferase (ASH1L) knockout on the relative expression levels of CTR1/α-tubulin protein (normalized band gray value results) in A498-DR cells. Left panel: cycloheximide (CHX) treatment (0, 6, 12, 24 h); Right panel: Baf-A1 treatment (0, 1, 8, 16 h). sgNC: negative control group; sgASH1L: ASH1L knockout group. Data are presented as mean ± standard deviation, analyzed by *t*-test (n = 3); *p < 0.05, **p < 0.01, ***p < 0.001.

In subcellular localization experiments (Fig. 6G), wheat germ agglutinin (WGA) was used to label cell membranes and LysoTracker to label lysosomes. It was observed that after ASH1L knockdown, the distribution of CTR1 in subcellular structures such as lysosomes changed. To investigate the effect of ASH1L on CTR1 protein stability, experiments with the protein synthesis inhibitor cycloheximide (CHX) were conducted (Fig. 6H and Fig. A11). The results showed that after ASH1L knockdown, the degradation rate of CTR1 was significantly slowed down. In contrast, experiments with the lysosomal inhibitor bafilomycin A1 (BaF-A1) (Fig. 6I and Fig. A10) indicated that ASH1L might regulate the degradation process of CTR1 through the lysosomal pathway.

**Figure A12:**
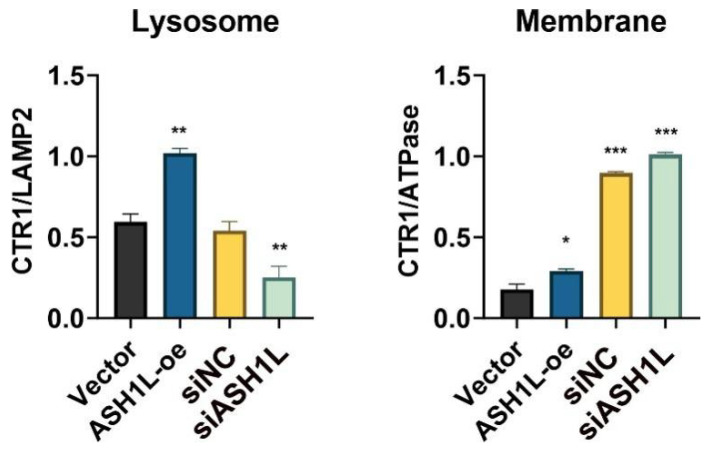
Effect of ASH1L regulation on the relative expression levels of CTR1 protein in lysosome (normalized to LAMP2) and membrane (normalized to ATPase) fractions. Vector: empty vector control group; ASH1L-oe: ASH1L overexpression group; sgNC: negative control group; sgASH1L: ASH1L knockout group. Data are presented as mean ± standard deviation, analyzed by *t*-test (n = 3); *p < 0.05, **p < 0.01, ***p < 0.001.

In addition, detection of the lysosomal membrane protein LAMP2 and the cell membrane protein Na^+^/K^+^ ATPase (Fig. 6J and Fig. A12) revealed that overexpression of ASH1L affected the distribution of CTR1 in structures such as lysosomes. At the molecular structure level, Molecular docking experiments demonstrated the interaction mode of ASH1L and CTR1 protein molecules in three-dimensional space (Fig. 6K), intuitively presenting the key structural regions and possible binding sites HIS-132, GLU-2273, ASN-2256, TYR-147, ASP2199, TRY-156) for their binding, and providing a visual theoretical reference for explaining the interaction between ASH1L and CTR1 from the perspective of molecular structure.

**Figure A13:**
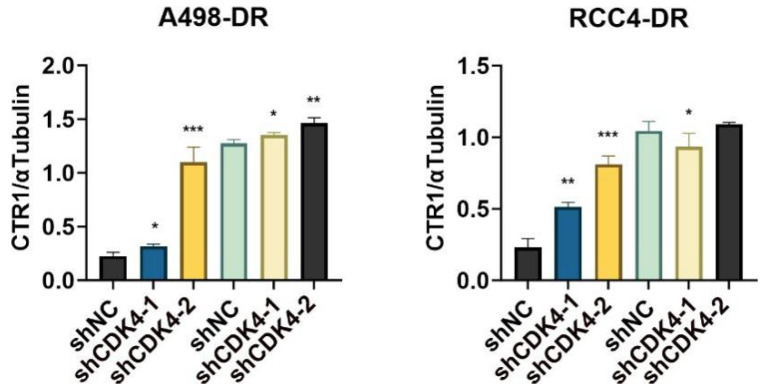
Effect of CDK4 knockdown on the relative expression levels of CTR1/α-Tubulin protein (normalized band gray value results) in A498-DR and RCC4-DR cells. shNC: negative control group; shCDK4-1, shCDK4-2: CDK4 knockdown groups (two interference sequences). Data are presented as mean ± standard deviation, analyzed by *t*-test (n = 3); *p < 0.05, **p < 0.01, ***p < 0.001.

**Figure A14:**
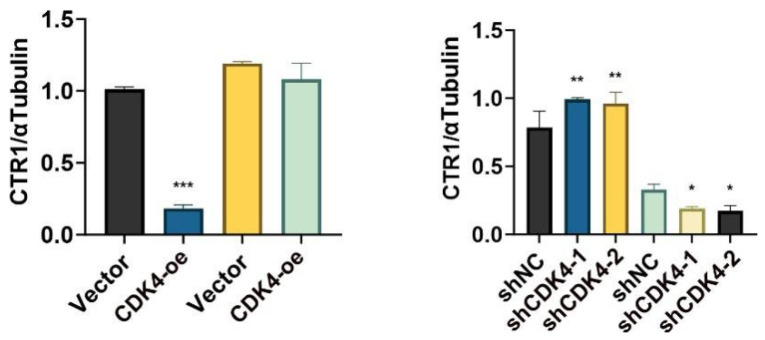
Effect of CDK4 overexpression and knockdown on the relative expression levels of CTR1/α-Tubulin protein (normalized band gray value results) in cells. Left panel: Vector: empty vector control group; Cdk4-oe: CDK4 overexpression group. Right panel: shNC: negative control group; shCDK4-1, shCDK4-2: CDK4 knockdown groups (two interference sequences). Data are presented as mean ± standard deviation, analyzed by *t*-test (n = 3); *p < 0.05, **p < 0.01, ***p < 0.001.

Finally, experiments involving knockout or overexpression of related genes (Fig. 6L–N, Fig. A13 and Fig. A14) further verified that the regulatory effect of ASH1L can independently regulate the expression and degradation processes of CTR1, which further clarifies the core role of ASH1L in regulating CTR1. In conclusion, ASH1L interacts with CTR1, and ASH1L mainly regulates CTR1 protein levels by affecting its lysosomal pathway-mediated degradation.

### 3.7 Regulation of Renal Cell Carcinoma Growth and Related Gene Expression by CDK4, ASH1L, and Chemotherapeutic Drugs

To further explore the roles of CDK4, ASH1L, and the chemotherapeutic drugs cisplatin (CIS) and gemcitabine (GEM) in the occurrence, development, and related gene regulation of RCC, a series of experiments was conducted.

**Figure 7:**
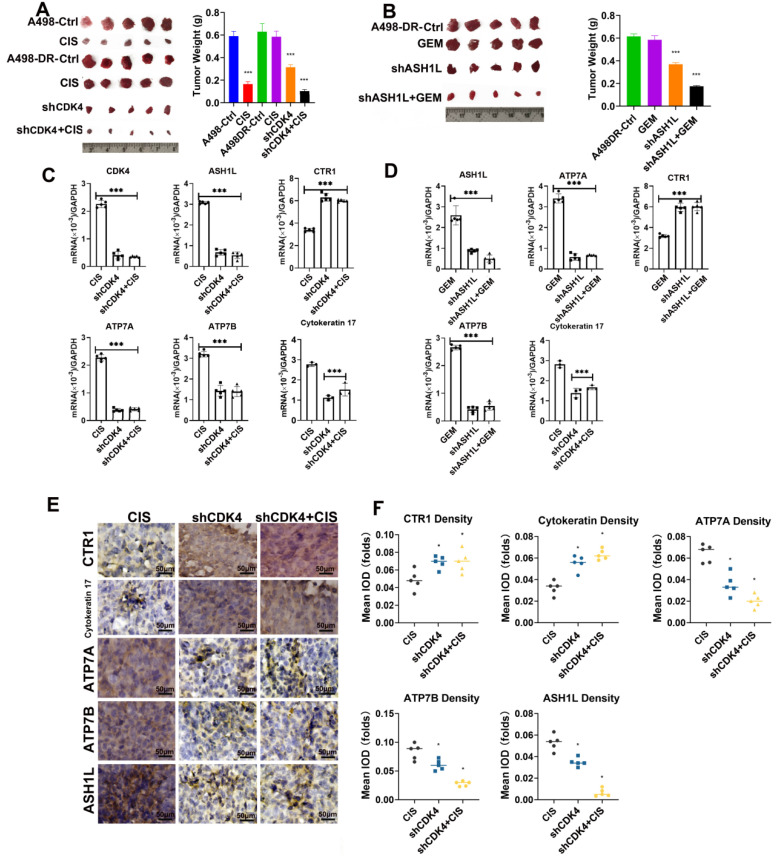
**Effects of CDK4/ASH1L-Related Interventions on RCC Tumors and Molecular Expression.** (**A**): Photographs and weights of tumors after intervention with cisplatin (CIS) and shCDK4; (**B**): Photographs and weights of tumors after intervention with gemcitabine (GEM) and shASH1L; (**C**,**D**): mRNA levels of related genes; (**E**): Immunohistochemical staining, bar: 50 μm; (**F**): Statistical analysis of the density of each molecule. (One-way ANOVA), *p < 0.05; ***p < 0.001.

First, *in vivo* tumorigenesis experiments using A498-DR cells (Fig. 7A,B) showed that in the RCC group treated with CDK4 knockdown combined with CIS, tumor weight was significantly lower than that in the control group (Ctrl) and the group treated with CIS alone, confirming that CDK4 knockdown enhances the sensitivity of cisplatin-resistant cell lines to cisplatin. Similarly, in the RCC group treated with ASH1L knockdown combined with GEM, tumor weight was also significantly lower than that in the control group and the group treated with GEM alone. This indicates that knockdown of CDK4 or ASH1L can enhance the anti-tumor effects of CIS and GEM on RCC.

Subsequently, qPCR was used to detect the expression of related genes (Fig. 7C,D). The results showed that after RCC was treated with CDK4 or ASH1L knockdown combined with the corresponding drugs, the expression levels of CDK4 and ASH1L genes were significantly downregulated, while the expression of genes such as CTR1, ATP7A, ATP7B, and Cytokeratin 17 also showed corresponding changing trends. This suggests that these genes may be involved in the regulatory process of RCC mediated by CDK4, ASH1L, and chemotherapeutic drugs. Finally, verification was performed at the protein level using immunohistochemical experiments (Fig. 7E,F). The results showed that after different treatments, the expression levels of proteins such as CTR1, Cytokeratin 17, ATP7A, ATP7B, and ASH1L in RCC changed. Statistical analysis of the mean optical density (Mean OD Density) further supported this conclusion, indicating that CDK4, ASH1L, and the chemotherapeutic drugs CIS and GEM can affect the progression of RCC by regulating the expression of the aforementioned genes and proteins.

## 4 Discussion

Cisplatin resistance remains a critical bottleneck in the clinical management of advanced RCC, leading to treatment failure and poor patient outcomes [8,9]. Accumulating evidence highlights the role of cyclin-dependent kinases (CDKs) in tumor progression and drug resistance, but the specific function of CDK4 in RCC cisplatin resistance has not been fully elucidated [10,11,12,13]. The present study systematically investigated the biological role and molecular mechanism of CDK4 in RCC cisplatin resistance through clinical sample analysis, *in vitro* cell experiments, and *in vivo* animal models, and identified the CDK4-ASH1L-CTR1 axis as a key regulatory pathway mediating this process.

### 4.1 CDK4 Is a Prognostic Biomarker and Key Driver of Cisplatin Resistance in RCC

Clinical sample analysis is the foundation of translating basic research to clinical practice. Our IHC results demonstrated that CDK4 expression was barely detectable in normal renal tissues but significantly upregulated in cisplatin-sensitive RCC tissues, with the highest expression in cisplatin-resistant (Tumor-DR) tissues (Fig. 1A,B). Survival analysis further confirmed that high CDK4 expression was associated with shorter overall survival (OS) and progression-free survival (PFS) in both cisplatin-sensitive and resistant RCC patients (Fig. 1C,D), with hazard ratios of 1.75 (*p* = 0.005) and 1.39 (*p* = 0.032), respectively. These findings are consistent with the pro-tumor role of CDK4 in breast cancer and lung cancer [14,15,16,17], and further extend its function to RCC cisplatin resistance, suggesting that CDK4 could serve as a potential prognostic biomarker for RCC patients receiving cisplatin chemotherapy.

*In vitro* functional experiments using parental RCC cells (A498, RCC4) and their cisplatin-resistant sublines (A498-DR, RCC4-DR) provided direct evidence for the role of CDK4 in cisplatin resistance. We first confirmed that A498-DR and RCC4-DR cells exhibited stronger cisplatin tolerance, proliferation, and migration abilities compared to parental cells, which was accompanied by significantly increased CDK4 expression (Fig. 2A–I, Table A4). Gain-of-function and loss-of-function assays further validated that CDK4 overexpression reduced cisplatin sensitivity and enhanced the malignant phenotypes (proliferation, migration) of parental RCC cells, while CDK4 knockdown in resistant cells reversed cisplatin resistance and suppressed tumorigenic potential (Fig. 3A–G). These results are consistent with the *in vivo* findings: CDK4 knockdown significantly inhibited tumor growth in nude mice xenografted with A498-DR cells and enhanced cisplatin sensitivity without obvious systemic toxicity (Fig. 4A–J). Collectively, these data from clinical samples, *in vitro*, and *in vivo* experiments collectively confirm that CDK4 is not only a prognostic indicator but also a key driver of cisplatin resistance and malignant progression in RCC.

### 4.2 The CDK4-ASH1L-CTR1 Axis Mediates Cisplatin Resistance via Transcriptional and Post-Translational Regulation

To explore the molecular mechanism underlying CDK4-mediated cisplatin resistance, we employed transcriptome sequencing, CUT&Tag, ChIP, and dual-luciferase reporter assays. Transcriptome analysis of CDK4-knockout A498-DhiR cells identified ASH1L (a histone methyltransferase) as a potential downstream target of CDK4 (Fig. 5A–E). Subsequent qPCR and Western blot results confirmed that CDK4 knockdown significantly reduced ASH1L mRNA and protein expression in multiple RCC-resistant cell lines (Fig. 5G–I). ChIP and dual-luciferase reporter assays further revealed that CDK4 directly binds to the promoter region of ASH1L (especially binding sites 2 and 3) to promote its transcription, as mutation of these sites abrogated the regulatory effect of CDK4 on ASH1L promoter activity (Fig. 5J–L). This finding expands the non-canonical function of CDK4 beyond cell cycle regulation [12,13], demonstrating its role as a transcriptional regulator in RCC.

ASH1L has been reported to be involved in tumor drug resistance by regulating histone modification [23,24], but its interaction with copper transporters in cisplatin resistance remains unclear. Our study found that ASH1L knockdown significantly upregulated CTR1 protein expression in RCC-resistant cells (Fig. 6A), while immunofluorescence co-localization, Co-IP, and GST-pull down assays confirmed a direct protein-protein interaction between ASH1L and CTR1 (Fig. 6B–F). CTR1 is a key copper transporter that mediates cisplatin uptake into cells, and its downregulation is closely associated with cisplatin resistance [25,26,27,28]. We further validated that CTR1 downregulation by siRNA attenuated cisplatin-induced growth inhibition in parental RCC cells, while CTR1 overexpression reversed cisplatin resistance in A498-DR and RCC4-DR cells (Fig. A6 and Fig. A7), consistent with previous reports [28,29].

Mechanistically, CHX chase and bafilomycin A1 (lysosomal inhibitor) experiments demonstrated that ASH1L regulates CTR1 protein stability via the lysosomal degradation pathway: ASH1L knockdown slowed CTR1 degradation, while lysosomal inhibition abrogated this effect (Fig. 6H,I). Subcellular localization analysis further showed that ASH1L overexpression altered CTR1 distribution in lysosomes and cell membranes (Fig. 6G,J), which may affect CTR1-mediated cisplatin uptake. Molecular docking simulations visualized the three-dimensional binding mode of ASH1L and CTR1, identifying key interaction sites (HIS-132, GLU-2273, ASN-2256, etc.) that may be critical for regulating CTR1 stability (Fig. 6K). These results collectively reveal a novel post-translational regulatory mechanism by which ASH1L promotes CTR1 lysosomal degradation, thereby reducing cisplatin uptake and inducing resistance.

### 4.3 Targeting the CDK4-ASH1L-CTR1 Axis Enhances Chemotherapeutic Efficacy in RCC

**Figure A15:**
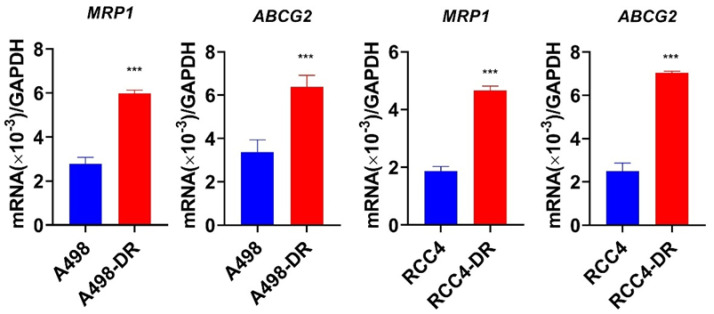
mRNA expression levels of MRP1 and ABCG2 in parental cells and cisplatin-resistant cells. Blue bars represent parental cells (A498/RCC4), and red bars represent resistant cells (A498-DR/RCC4-DR). Data are presented as mean ± standard deviation, n = 5, ***p < 0.001 (vs. parental cells).

The ultimate goal of exploring drug resistance mechanisms is to develop effective therapeutic strategies. Results from Fig. A15 revealed that the mRNA expression levels of MRP1 and ABCG2 were significantly upregulated in the resistant lines, suggesting that their overexpression is one of the molecular mechanisms underlying cisplatin resistance. Our *in vivo* experiments confirmed that targeting CDK4 or ASH1L could enhance the anti-tumor efficacy of chemotherapeutic drugs. Specifically, CDK4 knockdown combined with cisplatin (CIS) significantly reduced tumor weight and volume in A498-DR xenografts compared to CIS monotherapy (Fig. 7A), while ASH1L knockdown synergized with gemcitabine (GEM) to inhibit tumor growth (Fig. 7B). These findings are supported by molecular level changes: qPCR and IHC results showed that CDK4/ASH1L knockdown combined with chemotherapy downregulated CDK4 and ASH1L expression, while upregulating CTR1 (Fig. 7C–F). Additionally, changes in the expression of ATP7A, ATP7B, and Cytokeratin 17 (Fig. 7E,F) suggest that these molecules may be involved in the CDK4-ASH1L-CTR1 regulatory network, which warrants further investigation.

Notably, gemcitabine is a commonly used second-line chemotherapeutic drug for advanced RCC [3], and our results show that ASH1L knockdown enhances its anti-tumor effect, providing a theoretical basis for combination therapy strategies. These findings are consistent with previous studies showing that CDK4/6 inhibitors can synergize with chemotherapy to improve treatment outcomes [11,16], and further suggest that combining CDK4/ASH1L inhibitors with cisplatin or gemcitabine may be a promising therapeutic approach for cisplatin-resistant RCC.

### 4.4 Limitations and Future Directions

Despite the comprehensive findings, this study has certain limitations. First, the clinical sample size of cisplatin-resistant RCC was relatively small, and multi-center, large-sample studies are needed to validate the prognostic value of CDK4. Second, although we confirmed the role of the CDK4-ASH1L-CTR1 axis in A498 and RCC4 cell lines, the regulatory effect of this axis in other RCC subtypes (e.g., chromophobe carcinoma) remains to be explored.

In conclusion, this study demonstrates for the first time that CDK4 promotes cisplatin resistance in RCC by regulating the ASH1L-CTR1 axis: CDK4 directly binds to the ASH1L promoter to enhance its transcription, and ASH1L interacts with CTR1 to induce its lysosomal degradation, thereby reducing cisplatin uptake and mediating resistance. Clinically, CDK4 can serve as a prognostic biomarker for RCC patients receiving cisplatin chemotherapy. Therapeutically, targeting CDK4 or ASH1L may reverse cisplatin resistance and enhance the efficacy of cisplatin or gemcitabine, providing new strategies for the treatment of advanced cisplatin-resistant RCC.

## Data Availability

The datasets used and analyzed during the current study are available from the corresponding author on reasonable request.

## Appendix A

**Table A1:** Complete Baseline Clinical Information and Follow-Up Data of Included RCC Patients.

Clinical Characteristics	Total (n = 182)	Cisplatin-Sensitive Group (Tumor Group, n = 153)	Cisplatin-Resistant Group (Tumor-DR Group, n = 29)	*p* Value
1. Demographic Characteristics	-	-	-	-
Age (years)	-	-	-	-
- Median age (range)	58.5 (32–79)	57.2 (32–78)	62.1 (41–79)	0.132
- <60 years, n (%)	101 (55.5)	88 (57.5)	13 (44.8)	0.217
- ≥60 years, n (%)	81 (44.5)	65 (42.5)	16 (55.2)	-
Gender	-	-	-	-
- Male, n (%)	112 (61.5)	95 (62.1)	17 (58.6)	0.703
- Female, n (%)	70 (38.5)	58 (37.9)	12 (41.4)	0.622
2. Tumor Pathological Characteristics	-	-	-	-
AJCC Stage (8th Edition)	-	-	-	<0.001
- Stage I, n (%)	42 (23.1)	40 (26.1)	2 (6.9)	-
- Stage II, n (%)	58 (31.9)	55 (35.9)	3 (10.3)	-
- Stage III, n (%)	61 (33.5)	48 (31.4)	13 (44.8)	-
- Stage IV, n (%)	21 (11.5)	10 (6.5)	11 (37.9)	-
Fuhrman Nuclear Grade	-	-	-	<0.001
- Grade I, n (%)	28 (15.4)	27 (17.6)	1 (3.4)	-
- Grade II, n (%)	75 (41.2)	70 (45.8)	5 (17.2)	-
- Grade III, n (%)	59 (32.4)	46 (30.1)	13 (44.8)	-
- Grade IV, n (%)	20 (10.9)	10 (6.5)	10 (34.5)	-
Pathological Subtyp	-	-	-	0.085
- Clear Cell Carcinoma, n (%)	156 (85.7)	132 (86.3)	24 (82.8)	-
- Papillary Carcinoma, n (%)	18 (9.9)	15 (9.8)	3 (10.3)	-
- Chromophobe Carcinoma, n (%)	8 (4.4)	6 (3.9)	2 (6.9)	-
3. Previous Cisplatin Treatment Regimen	-	-	-	-
Cisplatin Dose (mg/m^2^), median (range)	-	-	75 (60–80)	-
Number of Cisplatin Cycles	-	-	-	-
- 2 cycles, n (%)	-	-	5 (17.2)	-
- 3–4 cycles, n (%)	-	-	21 (72.4)	-
- ≥5 cycles, n (%)	-	-	3 (10.3)	-
Combined Medication	-	-	-	-
- Cisplatin Monotherapy, n (%)	-	-	8 (27.6)	-
- Cisplatin + Gemcitabine, n (%)	-	-	17 (58.6)	-
- Cisplatin + Others (e.g., Sunitinib), n (%)	-	-	4 (13.8)	-
4. Follow-Up Data	-	-	-	-
- Median Follow-Up Duration, months (months)	36.2 (6.5–62.8)	37.5 (8.2–62.8)	31.8 (6.5–58.4)	0.205
Overall Survival (OS)	-	-	-	<0.001
- Median OS, months (95% CI)	48.6 (42.1–55.2)	52.3 (46.8–57.9)	29.5 (22.3–36.7)	-
- 1-Year OS Rate, % (95% CI)	92.3 (88.1–96.5)	95.4 (92.1–98.7)	75.9 (60.2–91.6)	-
- 3-Year OS Rate, % (95% CI)	78.6 (72.5–84.7)	85.6 (80.1–91.1)	44.8 (26.5–63.1)	-
Disease-Free Survival (DFS)	-	-	-	<0.001
- Median DFS months (95% CI)	39.8 (34.5–45.1)	45.2 (39.8–50.6)	18.7 (12.4–25.0)	-
- 1-Year DFS Rate, % (95% CI)	88.5 (83.6–93.4)	94.1 (90.2–98.0)	58.6 (40.1–77.1)	-
- 3-Year DFS Rate, % (95%CI)	69.2 (62.7–75.7)	78.3 (72.1–84.5)	27.6 (11.9–43.3)	-

**Table A2:** Information of sequences for gene silencing.

Primer Symbol	Primer Direction	Sequences (5′to 3′)	Company
*ShNC (ASH1L)* *ShASH1L* *ShNC (CDK4)* *ShCDK4-1* *ShCDK4-2* *SiNC (CDK4)* *SiCDK4-1*	N/A	CCGGTTCTCCGAACGTGTCACGTT	GenePharma, Suzhou, China
	N/A	TCAAGAGAACGTGACACGTTCGGA	
	sense	CCGGATCGTAGCTAGCATTAGCTATC	
	antisense	CCGGATCATTATCCGCGCATTCATCA	
	N/A	CCGGTTCTCCGAACGTGTCACGTTT	
	N/A	CAAGAGAACGTGACACGTTCGGAG	
	sense	CCGGACCGATTCTATCGGCATAGATC	
	antisense	AATTAAAAAACCGATTCTATCGGCAT	
	sense	CCGGACGAGAGCTTTCGAGCGAGA	
	antisense	AATTAAAAAACGAGAGCTTTCGAG	
	N/A	CCGGTTCTCCGAACGTGTCACGTTT	
	N/A	CAAGAGAACGTGACACGTTCG	
	sense	ACCGATTCTATCGGCATAGATCAT	
	antisense	TCCGATAGATCGATAGACTAGACA	
*SiCDK4-2*	sense	ACGAGAGCTTTCGAGCGAGACTA	
	antisense	TTAGGTTACATAGCGCGATATAAT	
*SiNC (ASH1L)* *SiASH1L-1*	N/A	CCGGTTCTCCGAACGTGTCACGTT	
	N/A	CAAGAGAACGTGACACGTTCGG	
	sense	ATCGTAGCTAGCATTAGCTATCGA	
	antisense	TAGCTACGGCTACTTACATACACT	
*SiASH1L-2*	sense	ATCATTATCCGCGCATTCATCAGG	
	antisense	TAGCCCGATAGATTCGTACGTCCC	

**Table A3:** Sequence information of relevant primers.

Primer Name	Primer Sequence (5′ to 3′)
MRP-1-F	GCGAGTGTCTCCCTCAAACG
MRP-1-R	TCCTCACGGTGATGCTGTTC
ABCG2-F	GCTGTCATGGCTGGACTTCA
ABCG2-R	TGGACATCATAGGCAAGGAGAA
ASH1L-F	GCTGCTGATGCTGATGATGA
ASH1L-R	TCTGCTGCTGCTGCTGCT
CTR1-F	ATGGCCTTCCTGCTGCTG
CTR1-R	GCTGCTGCTGCTGCTGCTG
ATP7A-F	GCTGCTGATGCTGATGATGA
ATP7A-R	TCTGCTGCTGCTGCTGCT
ATP7B-F	ATGGCCTTCCTGCTGCTG
ATP7B-R	GCTGCTGCTGCTGCTGCTG
Cytokeratin 17-F	GGTGGGTGGTGAGATCAATGT
Cytokeratin 17-R	CGCGGTTCAGTTCCTCTGTC
CDK4-F	GCTGCGAAGTCGTGTGAAGT
CDK4-R	CAGGGTCACCTTCTTCACCA
GAPDH-F	AGGAGCTGCTGAAGATGGAC
GAPDH-R	TCCTTGGTCTCGGAGTTGGT
ASH1L-F	GAAGGTGAAGGTCGGAGTC
ASH1L-R	GAAGATGGTGATGGGATTTC

**Table A4:** Comparison of IC_50_ Values, Resistance Indices, and Passage Stability between Parental Cells and Cisplatin-Resistant Cells (A498-DR/RCC4-DR).

Cell Line	IC_50_ of Parent Cells (μg/mL), Mean ± SD	IC_50_ of Resistant Cells (μg/mL), Mean ± SD	Resistance Index (RI)	IC_50_ after 20 Passages (μg/mL), Mean ± SD	RI Change Rate after Passage
A498/A498-DR	1.23 ± 0.15	18.76 ± 1.24	15.25	17.92 ± 0.98	4.0% (no significant difference)
RCC4/RCC4-DR	1.85 ± 0.21	22.34 ± 1.56	12.08	21.57 ± 1.13	3.4% (no significant difference)

Note: Abb: IC_50_: half maximal inhibitory concentration, SD: standard deviation, DR: drug-treated, RCC: renal cell carcinoma.
